# Conformation-driven quantum interference effects mediated by through-space conjugation in self-assembled monolayers

**DOI:** 10.1038/ncomms13904

**Published:** 2016-12-20

**Authors:** Marco Carlotti, Andrii Kovalchuk, Tobias Wächter, Xinkai Qiu, Michael Zharnikov, Ryan C. Chiechi

**Affiliations:** 1 Stratingh Institute for Chemistry & Zernike Institute for Advanced Materials, University of Groningen, Nijenborgh 4, Groningen 9747 AG, The Netherlands; 2 Applied Physical Chemistry, Heidelberg University, Im Neuenheimer Feld 253, Heidelberg 69120, Germany

## Abstract

Tunnelling currents through tunnelling junctions comprising molecules with cross-conjugation are markedly lower than for their linearly conjugated analogues. This effect has been shown experimentally and theoretically to arise from destructive quantum interference, which is understood to be an intrinsic, electronic property of molecules. Here we show experimental evidence of conformation-driven interference effects by examining through-space conjugation in which π-conjugated fragments are arranged face-on or edge-on in sufficiently close proximity to interact through space. Observing these effects in the latter requires trapping molecules in a non-equilibrium conformation closely resembling the X-ray crystal structure, which we accomplish using self-assembled monolayers to construct bottom-up, large-area tunnelling junctions. In contrast, interference effects are completely absent in zero-bias simulations on the equilibrium, gas-phase conformation, establishing through-space conjugation as both of fundamental interest and as a potential tool for tuning tunnelling charge-transport in large-area, solid-state molecular-electronic devices.

## Abstract (web summary)

Tunnelling currents through molecular junctions are affected by quantum interference effects, but understanding the factors leading to them remains a challenge. Here the authors show that through-space conjugation in self-assembled monolayers leads to conformation-dependent quantum interference that suppresses conductivity.

## Graphical abstract

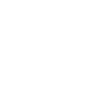

A central challenge in molecular electronics is uncertainty in the conformation and binding geometry of a molecule between two electrodes. Small differences exert large effects on tunnelling charge-transport^1^, yet it is this sensitivity that drives scientific interest because functionality arises from precise control over conformation and geometry^2^,^3^,^4^. Nature accomplishes this level of control through self-assembly, in which molecular systems can be arranged precisely enough to support long-range electron transfer over several microns^5^. In self-assembled monolayers (SAMs), molecules are fixed in a specific conformation and binding geometry, arranging themselves in ordered, two-dimensional crystal-like domains. Tunnelling junctions comprising SAMs, therefore, fix molecules in a specific conformation and binding geometry that defines the smallest dimension of the junction, through which charges tunnel; they are a form of bottom-up nanotechnology^6^,^7^. The effects of conformational confinement on molecular charge-transport are particularly interesting in the case of *π*-conjugated molecules because conductivity (hopping) and transmission (tunnelling) are strongly related to the extent of electronic delocalization. Quantum interference (QI) effects^8^ arising from cross-conjugation in such conjugated molecules have been demonstrated experimentally^9^,^10^, but they have also been predicted for *π* systems that are close enough in space to interact^11^. Unlike for conjugation patterns, which are an intrinsic property, this type of QI is extremely sensitive to conformation; the two *π* systems must be precisely aligned to form a through-space conjugation interaction.

Single-molecule conductance techniques are not well suited for observing effects that require precise control over conformation because they apply force to molecules and the geometry of the junction differs with each observation^12^,^13^. And although SAMs are crystalline and can be highly ordered over small areas, defects and grain boundaries can influence the current-density versus voltage (*J*/*V*) properties in large-area (μm^2^) measurements^14^. In addition to these experimental challenges, distinguishing between destructive QI and differences in conductance arising from conjugation length require fortuitous level-alignment in the assembled junction^15^. Through-space QI is potentially useful beyond validating theoretical predictions as it could couple small structural or conformational changes (for example, from external mechanical forces) into exponential changes in conductance. It is also synthetically advantageous because cross-conjugation tends to involve heteroatoms, which can introduce unexpected and complex electrostatic effects^16^.

Here we describe through-space destructive QI effects in tunnelling charge-transport through SAMs comprising molecules in which the central aromatic rings are spatially separated by saturated methylene bridges. These effects are consistently present in simulated transmission spectra derived from the X-ray crystal structures of the compounds used to form the SAMs, but not in minimized, gas-phase geometries. We resolved these effects experimentally by conducting-probe atomic force microscopy (CP-AFM) and in large-area junctions using eutectic Ga-In (EGaIn) as a top-contact^17^. These results prove that the *J*/*V* characteristics are not the result of defects and demonstrate both that a single conformation resembling that of the bulk crystal dominates transport in a SAM, mitigating the problem of uncertainty in the geometry of tunnelling junction, and that symmetric Au contacts can be used to model electronic effects qualitatively in large-area junctions with EGaIn top-contacts.

## Results

### Conformation within the SAM

**Figure 1:**
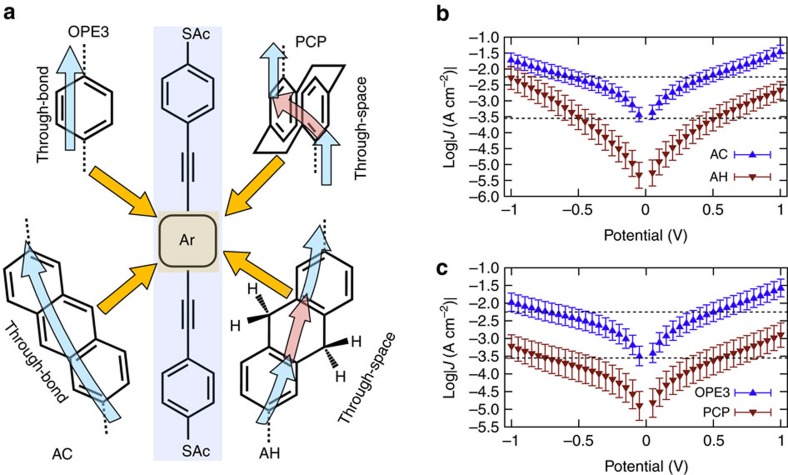
Molecular conformation effects on charge transport in tunnelling junctions. (**a**) The structures of the compounds from which SAMs were grown on Au^TS^. The blue arrows show through-bond pathways and the red arrows through-space pathways. (**b**) Current-density versus voltage plots of Au^TS^/SAM//EGaIn junctions of AH (red triangles) and AC (blue triangles) and (**c**); PCP (red triangles) and OPE3 (blue triangles). The dashed lines are to guide the eyes. Each data point is the peak of a Gaussian fit of log-normal plots of |*J*| for that voltage. Error bars are confidence intervals. Tunnelling junctions were formed by contacting grounded Au^TS^/SAM substrates with sharp tips of EGaIn and applying a potential.

We investigated tunnelling transport in systems characterized by two phenyl rings in either a vertical, face-on (*pseudo-p*-bis((4-(acetylthio)phenyl)ethynyl)-*p*-[2,2]cyclophane, PCP) or edge-on (2,6-bis(((4-acetylthio)phenyl)ethynyl)-9,10-dihydroantracene, AH) arrangement and that are held in close proximity with saturated ethylene bridges in Au^TS^/SAM//EGaIn and Au^M^/SAM//Au^AFM^ junctions (where ‘/' and ‘//' denote covalent and van der Waals contacts, respectively, Au^TS^ is template-stripped Au, Au^M^ is Au-on-mica and Au^AFM^ is a Au-coated AFM tip.) In both cases the precise alignment of the *π* systems is controlled by packing in the SAM on the Au substrates. We measured 1,4-bis(((4-acetylthio)phenyl)ethynyl)benzene (OPE3) and 2,6-bis(((4-acetylthio)phenyl)ethynyl)antracene (AC)^18^ to compare molecules with the same end-to-end lengths as PCP and AH, but that are conjugated through-bond; the structures and tunnelling pathways are shown in Fig. 1. Edge-on transport is difficult to address experimentally because at room temperature AH is in rapid equilibrium between bent and planar conformers in solution and in the gas phase, preferring the bent conformation by 3.5 kcal mol^−1^ (see Supplementary Table 1 and Supplementary Fig. 1). Thus, the bent conformer would dominate when AH is trapped in a single-molecule junction. In the crystalline state, however, AH adopts a planar conformation to minimize the free volume. To establish experimentally the precise spacial arrangements of the phenyl rings comprising the through-space elements, we obtained the X-ray crystal structures of AH and PCP, which we denote as AH-crystal and PCP-crystal. These structures and data are shown in Supplementary Figs 2 and 3 and Supplementary Table 2.

We determined the tunnelling transport properties by comparing the magnitude of log|*J*| in Au^TS^/SAM//EGaIn junctions comprising SAMs of OPE3, PCP, AH and AC, which we grew from symmetric bis-thioacetates via *in situ* deprotection (see ‘Methods' section)^9^,^19^. We characterized the SAMs by ellipsometry, high-resolution X-ray photoelectron spectroscopy (HRXPS) and near-edge X-ray absorption fine structure (NEXAFS) spectroscopy. These data are summarized in Table 1 along with literature and benchmark values for SAMs of CH_3_(CH_2_)_17_S (SC18) for comparison. The spectra are shown in Supplementary Figs 4 and 5. The principal difference appears to be the tilt angles, which are slightly higher for Au^TS^ than Au^TM^, thus the SAMs are slightly thinner (except for AH), but still quite dense (on the order of 10^14^ molecules per cm^2^). The S 2p X-ray photoelectron spectra of the SAMs exhibited characteristic signals of thiolate and unbound SH/SAc groups, with much higher intensity of the latter signals, because of the differences in the attenuation^20^. These spectra suggest that the molecules are indeed assembled upright in the SAMs, with one of the terminal sulfur atoms bound to the substrate and another one exposed to the SAM-ambient interface, where it can be contacted by EGaIn or Au. Upright molecular orientation is also apparent from the NEXAFS spectra, which exhibited quite small but distinct linear dichroism corresponding to an average molecular tilt angle of ∼40°. In addition, the spectra exhibited the characteristic absorption resonances of OPE-like compounds (Supplementary Note 1), establishing the identity of the AC, AH, OPE3 and PCP films. Significantly, there were no traces of contamination, including a lack of the very pronounced resonance of carboxyl moieties (most frequent contamination), demonstrating the high purity of the prepared monolayers. Measuring well characterized, high-quality SAMs is paramount as, unlike top-down, single-molecule techniques (break junctions and so on) or few molecules techniques (conducting-probe AFM and so on.), EGaIn is a bottom-up, large-area technique^7^ and is therefore sensitive to the detailed structure of the SAM because it defines the physical shape of the junction and the EGaIn//SAM interface^21^,^22^,^23^.

### Charge-transport characteristics

In through-space conjugation two *π* systems are held close enough in space to interact without the aid of an underlying *σ* framework. Electronic overlap and, therefore, tunnelling charge-transport is extraordinarily sensitive to the conformation imposed by the junction geometry because there is no rigid framework to keep *π* systems aligned and their relative displacement is subject to interactions with neighbouring molecules. This sensitivity is why through-space QI effects are difficult to resolve experimentally; for example, destructive QI is predicted in stacked benzene rings, but only through specific pathways and precise arrangements of the two rings^11^. Previous break-junction measurements on *p*-2,2-cyclophane moieties (which are similar to PCP) probed tunnelling charge-transport perpendicular to the plane of the phenyl rings (that is, down the stack)^24^. And the conductance of AH was higher than AC in break-junctions, where the former exhibited a decrease in conductance with increasing electrode separation^10^. No QI was predicted or observed in either of these studies; that is, an electron tunnelling through space (the red arrows in Fig. 1a) is not itself a predictor of QI or of different magnitudes of tunnelling transport compared with their through-bond analogues.

**Figure 2:**
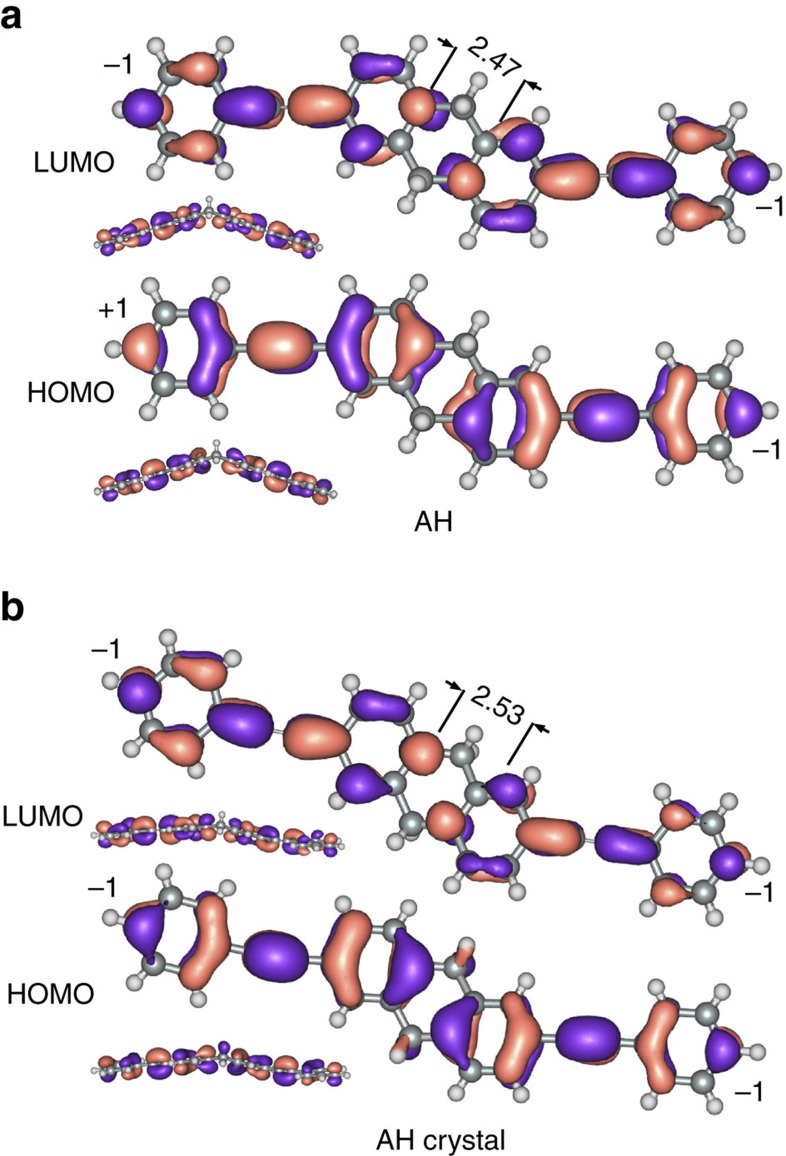
Impact of molecular geometry on molecular orbitals. The HOMO and LUMO of AH (**a**) and AH-crystal (**b**) with through-space distances indicated in Å. The signs of the orbital coefficients at the terminal sites are shown at these sites.

Figure 1b,c show the *J*/*V* curves for AC, AH, OPE3 and PCP; the histograms from which these data were derived are shown in Supplementary Fig. 6. The SAMs on Au^TS^ are robust enough to scan to ±1 V, revealing the onset of asymmetric conductivity (log|*J*|@1 V≠log|*J*|@−1 V) in AH, possibly because the two halves of AH approach resonance independently of each other due to the disparate contacts^25^. Data from Au^M^/SAM/Au^AFM^ junctions acquired using CP-AFM show the same trends in conductance, although we could not resolve the low-bias regions of PCP or AH (presumably because they are below the detection limit; Supplementary Figs 7 and 8.) Nonetheless, these data confirm that the trend is not due to defects in the SAM (the contact area of CP-AFM is on the order of dozens of molecules) or the influence of the Ga_2_O_3_ layer on the EGaIn electrode. Both the Au^M^/SAM/Au^AFM^ and Au^TS^/SAM/EGaIn junctions of OPE3 and AC exhibit larger magnitudes of *J* by about a factor of 100 than PCP and AH, respectively (which may be a sign that destructive QI is dominant in the latter two SAMs); however, unambiguously ascribing transport data to destructive QI effects is difficult because the observable is smaller magnitudes of log|*J*|, which is sensitive to myriad of factors^26^. Thus, we applied various tests for QI (see Supplementary Notes 2 and 3 and Supplementary Figs 9–16); it is predicted for PCP and AH, but not OPE3 or AC, in agreement with the *J*/*V* data in Fig. 1. One of these tests is the product of the coefficients of the frontier orbitals on the termini of a molecule; if they are the same, destructive QI is predicted between those orbitals^8^. This test is shown in Fig. 2 for AH and AH-crystal. The change in the signs of the orbital coefficients between AH and AH-crystal is accompanied by an increase in distance between the phenyl rings of 0.06 Å, at which point the two halves (separated by the CH_2_ bridges) become almost coplanar, aligning the two phenyl *π*-systems and allowing for better electronic overlap. In this conformation the two phenyl rings are connected by through-space conjugation and this form of through-space conjugation apparently induces QI analogously to the face-on arrangement in PCP; however, QI has not previously been predicted for AH because only the gas-phase minimized structure has been considered. Destructive QI may be ‘switched on' in by small changes in conformation.

## Discussion

For further insight into the predicted transport properties, we simulated transmission spectra for isolated molecules of AC, AH, OPE3 and PCP bound to Au electrodes. To isolate relative effects of molecular structure, we performed density function theory (DFT) calculations with 12-atom Au(111) clusters bound to the terminal sulfurs at hexagonal close-pack hollow sites. These calculations are not models of Au^TS^/SAM//EGaIn or Au^M^/SAM/Au^AFM^ junctions, rather they are computational experiments on idealized systems to isolate the effects of electronic structure and conformation on zero-bias transmission by using single molecules bound to small clusters of Au. There are numerous collective effects in SAMs that can affect the electrostatics and level-alignment that cannot be captured by single-molecule calculations^27^,^28^,^29^,^30^. Without detailed knowledge of the packing of the molecules in the SAM and atomistic detail of the Ga_2_O_3_ electrode and SAM//Ga_2_O_3_EGaIn interface, none of which are available, it is impossible to construct an accurate model capable of predicting these effects. The principal limitations of using a model Au/molecule/Au junction is that we cannot reliably estimate the Fermi level *E*_f_ and the relative level-alignments will not reflect any collective effects such as broadening or electrostatic shifts^31^ (for example, the 0.4–0.5 eV shift induced by S–Au bonds in SAMs^32^). However, we can still compare (similar) molecular structures and draw meaningful (qualitative) conclusions in combination with experimental data from Au^TS^/SAM//EGaIn junctions^9^. We estimated *E*_f_ by adding the calculated highest-occupied *π* state (HOPS) to the experimentally derived transition voltages^31^,^33^ *V*_trans_ for each Au^TS^/SAM//EGaIn junction (see Supplementary Table 3) to establish the relative level-alignment between related structures (that is, OPE3 and PCP; AH and AC).

**Figure 3:**
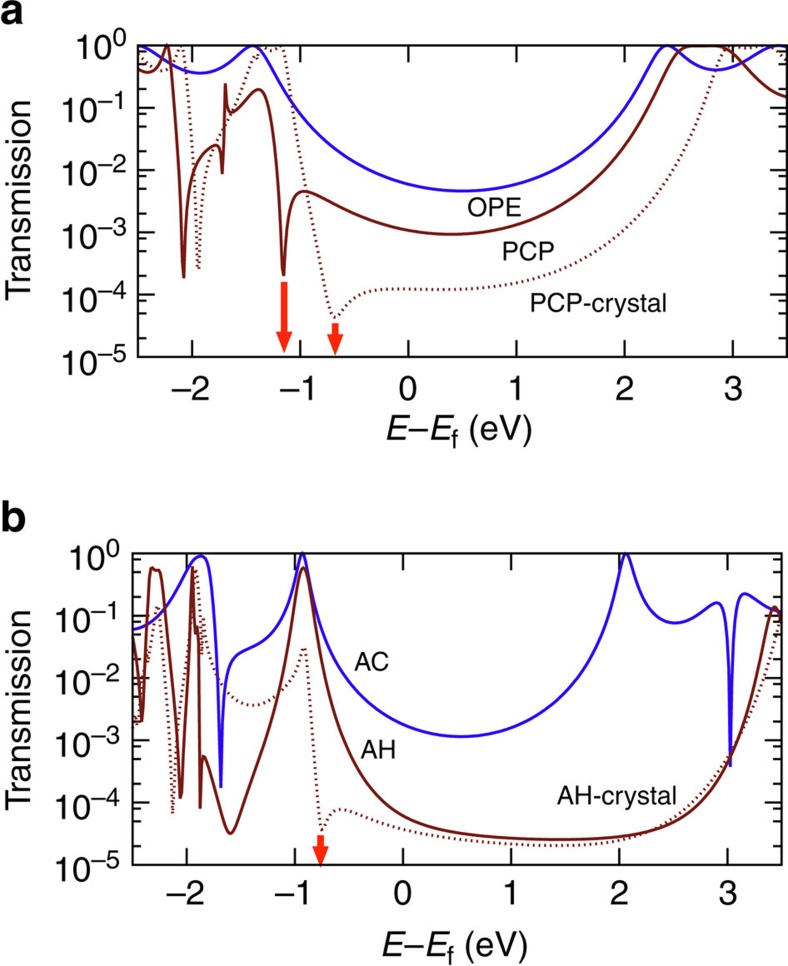
Charge transmission probability plots. Transmission plots comparing, (**a**) PCP (red), OPE3 (blue) and PCP-crystal (dashed red) and (**b**) AH (red), AC (blue) and AH-crystal. The ‘crystal' suffix denotes X-ray crystal structures; the others are gas-phase minimized structures. The energy of the Fermi level *E*_f_ was computed by adding *V*_trans_ to the HOPS. Destructive QI features are marked with thick red arrows.

Figure 3a shows the resulting transmission spectra from DFT calculations for OPE3 and PCP Au/molecule/Au junctions. The U-shape centred at ∼0.5 eV is indicative of transmission probabilities in the frontier orbital gap of the molecule. The lower overall transmission of and PCP and PCP-crystal is the result of the the break in conjugation at the cyclophane ring, that is, there is no formal resonance structure connecting the two halves of the molecule. The plots for PCP and PCP-crystal also exhibit sharp dips around −1 eV, which is a sign of QI; at this energy the frontier orbitals interfere destructively and a node appears in the wave function, suppressing transmission sharply. The most pronounced differences between PCP and PCP-crystal are that the transmission is higher everywhere for the former and the energy and magnitude of the dip ascribed to QI shifts by ∼0.5 eV (that is, the positions and lengths of the solid red arrows in Fig. 3a differ.) This observation highlights the utility and limitations of using zero-bias, single-molecule junctions as models for Au^TS^/SAM//EGaIn junctions; both the overall conductance (the integrated transmission near *E*_f_) and the position of the QI feature are sensitive to minor conformational changes. Supplementary Fig. 17 compares some of these differences, which are as small as 0.05 Å in the cyclophane core. The net effect is that the *x*-axes in Fig. 3 as well as the relative position of the dip in transmission will be shifted in the actual junctions.

Figure 3b shows the transmission spectra of AC and AH Au/molecule/Au junctions. Analagously to OPE3, AC shows a U-shaped curve centred at ∼0.5 eV; unlike OPE3 and PCP, however, the positive resonances (not QI) near the frontier orbitals for AC are closer to 0 eV than they are for AH because there is a more pronounced change in the frontier orbital gap between linear and through-space conjugation in the edge-on case (AH) than the face-on case (PCP.) It is tempting to ascribe this difference between the investigated systems to the lower conductivity of AH compared with AC—one is conjugated and one is not--whereas cyclophanes exhibit similar properties to phenyl rings (for example, functional groups direct identical pseudo *para*/*meta* substitution). Fig. 1b,c, however, show that SAMs of PCP are experimentally much less conductive than SAMs of OPE3 across the entire bias window, while AH approaches the conductivity of AC, nearly crossing at −1 V. This behaviour is consistent with hypothesis that the conductance of AH in the low-bias regime is dominated by a sharp, destructive QI feature and that, as the bias is increased, transmission increases rapidly; that is, the dip in the dashed line (AH-crystal) in Fig. 3b is shifted close to *E*_f_ in actual Au^TS^/SAM//EGaIn junctions. The solid line in Fig. 3b is the minimized, gas-phase conformation of AH; unlike the case of PCP and PCP-crystal, the interference feature present for AH-crystal vanishes entirely with AH. We ascribe this difference to the changes in the frontier orbitals shown in Fig. 2 when AH adopts a planar conformation in the solid-state. This comparison does not imply that AH-crystal reflects the exact conformation of AH in a SAM; however, if AH adopts a planar conformation in a bulk crystal, it is very likely that it does so when confined to a 2D molecular film on a solid substrate.

The extreme sensitivity of through-space QI effects on molecular conformation demonstrated by the DFT calculations presents a significant experimental challenge that has confounded past efforts to characterize the effects of through-space conjugation on tunnelling charge-transport. Wire-like *p*-2,2-cyclophane moieties similar to PCP probed by crossed-wires, scanning tunnelling microscopy and CP-AFM in mixed-monolayers and sparse SAMs—that is, not in dense SAMs like those formed from PCP—exhibited only slightly suppressed conductance^34^,^35^. And attempts to compare edge-on through-space and through-bond conduction pathways (analogous to AH) were similarly frustrated; although through-space interactions were found in the crystal structure, there was no correlation with conductance measurements in break-junctions^36^. The inconsistency of these observations may be a reflection of the sensitivity of through-space effects to conformation (or simply better experimental techniques). These results underscore the importance of considering the different geometries molecules adopt in solid, gas or dissolved phases and in single-molecule junctions and the challenges of growing densely packed SAMs from *π*-conjugated molecules.

**Figure 4:**
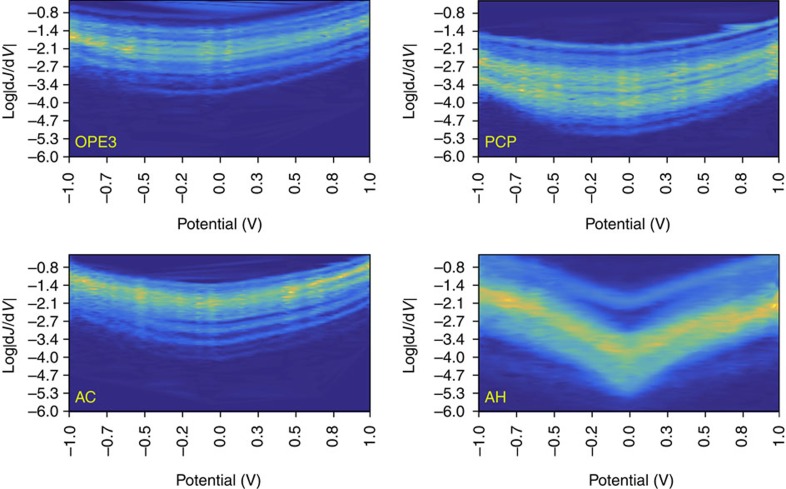
Differential conductance plots. Conductance heatmap plots of OPE3 and PCP (top left and right, respectively) and AC and AH (bottom left and right, respectively) showing histograms binned to log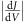 (conductance, *Y*-axis) versus potential (in *V*, *X*-axis). The colours correspond to the frequencies of the histograms; lighter colours indicate higher frequencies (max ∼150); the yellow bands are ∼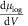 (that is, from Fig. 1). All of the plots except for AH are U-shaped, which is a sign of non-resonant tunnelling. The plot for AH has a sharp dip the centre and negative curvature near ±1 V, which is a sign that a destructive interference feature exists in the bias window.

We are confident in ascribing the low conductivity of SAMs of PCP to QI because it has been predicted for face-on through-space transport^11^, however, the most robust experimental proof of destructive QI is the appearance of negative curvature in conductance heatmap plots of differential conductance log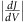 versus *V* (ref. 37). This curvature only appears if the energy of the interference in the assembled junction is close enough to *E*_f_ that the feature lies almost entirely within the bias window^15^. The dip in transmission for PCP and AH-crystal in Fig. 3 is far from *E*_f_, but (as described above) these calculations do not reflect the actual level-alignment in the Au^TS^/SAM//EGaIn junctions. Differential conductance heatmaps of OPE3, AC, PCP and AH are shown in Fig. 4. These plots are constructed from histograms of log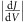 obtained from numerical derivatives of individual *J*/*V* plots. (One advantage of EGaIn measurements is that *J*/*V* curves do not require smoothing; these are ‘raw' derivatives.) The plots of OPE3, AC and PCP are all U-shaped and slightly asymmetric, reflecting non-resonant tunnelling with somewhat asymmetric coupling. This observation is consistent with recent work comparing Au/molecule/Au and Ag^TS^/SAM//EGaIn junctions^38^. The magnitudes of PCP and AH are also lower than OPE3 and AC, however, the AH curve is sharp with obvious negative curvature. This difference is direct, experimental evidence of a destructive QI feature very close to *E*_f_, supporting the hypothesis that AH in a SAM adopts a planar conformation closer to that of AH-crystal than to (the minimized, gas-phase conformation of) AH (Fig. 2).

Transport is dominated by states close to *E*_f_, but accurately determining the positions of these states in assembled tunnelling junctions is a principal experimental challenge. The only experimental observable for destructive QI is lower conductance, thus claims that QI leads to the observation that a particular structure is less conductive than a reference structure in tunnelling junctions rest entirely on theoretical methods such as those outlined in this paper. However, when the sharp resonance feature of destructive QI is sufficiently close to *E*_f_ (in physical junctions) it can appear as a V-shaped differential conductance plot. Next to this work, there are only two other reports of such features appearing, both involving cross-conjugated quinone derivatives of AC^37^,^39^. In both cases QI features close to *E*_f_ appear in DFT calculations for two structures, but appear in differential conductance plots for one. Thus, our work is the first experimental evidence of destructive QI modulated by through-space conjugation and the first observation of conformer-dependent QI. We base our claim that the interference feature AH of lies near *E*_f_ entirely on experimental evidence because it is not possible even to estimate the electrostatics in a metal/SAM/metal junction with the available atomistic and structural information; however, deriving *E*_f_ from *V*_trans_ gives eminently reasonable relative level-alignments between OPE3/PCP and AC/AH with respect to the HOPS. Given the similarity in length and HOPS between OPE3/PCP and AC/AH and the differential conductance plots, we can unambiguously ascribe the suppressed conductivity of both PCP and AH to the destructive QI in the transmission spectra of PCP-crystal and AH-crystal and conclude that *J* is dominated by a single conformer and that the through-space elements are better described by X-ray crystal structures than gas-phase optimized structures.

As the complexity of organic structures investigated in tunnelling junctions grows, the details of molecular conformations become more important. And moving from top-down spectroscopic tools towards functional, device-like platforms^2^,^3^,^4^,^40^,^41^,^42^,^43^,^44^ will probably involve bottom-up molecular tunnelling junctions based on SAMs^7^, in which molecules are in a (liquid) crystalline state. Such junctions represent a form of nanotechnology closest to Nature in that the nanoscopic structure and function are simultaneously and inseparably defined by the equilibrium self-assembly of molecules; differences of 0.06 Å–0.11 Å can completely suppress QI in DFT simulations. Using QI as a probe, we can separate the effects of interrupted *π* conjugation from those of QI, which are normally conflated experimental observables. And in doing so we provided unambiguous evidence that through-space conjugation can cause interference effects and are not simply ‘non-conjugated'. When detailed conformation and packing cannot be determined experimentally, X-ray crystal structures of the pure compounds may be a better approximation of molecular conformation in densely packed SAMs than DFT-minimized structures and transition voltages may provide a reasonable approximation for level-alignment to relate transmission calculations to SAM-based junctions. This approach to understanding transport properties should be generalizable—even in the absence of an observable such as through-space QI—facilitating detailed theoretical and experimental studies on bottom-up, large-area molecular junctions comprising SAMs.

## Methods

### Synthesis

The synthesis of AH, AC and OPE3 is described elsewhere^18^. All compounds were stored in nitrogen-flushed vials and in the dark. Their structures were verified by acquiring ^1^H-NMR and Fourier transform infrared spectra immediately before use and comparing with the spectra acquired immediately after purification. PCP was prepared starting from *p*-2,2-cyclophane as described in the Supplementary Methods and shown in Supplementary Fig. 18.

### Self-assembled monolayers

Care must be taken when forming SAMs from conjugated ‘molecular wire' compounds such as these because of the tendency for the deprotected dithiol(ates) to lie flat due to their bidentate structure and favourable *π*-Au interactions. SAMs of molecular wires on Au-on-mica were previously formed from a mixture of THF and Et_3_N (refs 9, 19), but in the current work Au^TS^ is used, which is supported by optical adhesive and is therefore incompatible with THF. Thus, the procedure was modified and SAMs were formed by incubating the thioacetate precursors with 1 × 1 cm template-stripped Au surfaces (100 nm-thick) overnight in 3 ml of 50 μM solution of the respective compound in freshly distilled toluene followed by addition of 0.05 ml of 17 mM diazabicycloundec-7-ene solution in toluene 1 h prior the measurement. The substrates were then rinsed with ethanol and let to dry for 10 min.

To minimize the chance of oxidative damage to the compounds and SAMs, sample preparation, handling and measurement with the EGaIn setup were all performed in a nitrogen flow box with a controlled O_2_ level between 1–3% (some O_2_ is necessary to form tips of EGaIn) and humidity below 10%. At least 20 junctions were measured on each of at least three substrates per molecule (10 scans from 0 V→1 V→−1 V→0 V, steps of 0.05 V) for a total of at least 600 traces per SAM. A new EGaIn tip was prepared every 5–8 junctions and flattened by gently pushing it on a Si wafer few times according to the procedure reported by Simeone *et al*.^26^. The details of the EGaIn setup are described elsewhere^9^.

Characterization of the SAMs was performed by HRXPS and NEXAFS spectroscopy. The measurements were carried our at the synchrotron storage ring MAX II at MAX-IV facility in Lund, Sweden and synchrotron radiation facility BESSY II in Berlin, Germany using the bending magnet beamlines D1011 and HE-SGM, respectively. HRXPS data were used to calculate the effective thickness and packing density of the SAMs. NEXAFS data were used to monitor the chemical identity and molecular inclination in the SAMs. See Supplementary Methods for details.

Ellipsometric measurements were acquired in air on a V-Vase Rotating Analyzer equipped with a HS-190 monochromator ellipsometer and calculated via a two-layer model consisting of a bottom Au layer, for which optical constants were calculated from freshly prepared template-stripped Au surfaces, and a Cauchy layer with a chosen value of *n*=1.5 and *k*=0 at all wavelengths (*A*=1.5, *B*=*C*=0)^19^.

### Calculations

All DFT calculations were performed using Orca 3.0.3 (ref. 45). Structures were first minimized using BP/Def2-TZV(2d) and then point energies were calculated using B3LYP/Def2-TZV(2d/sp). Single-molecule junctions were constructed by attaching the minimized or X-ray crystal structures to 12-atom Au(111) clusters via the terminal sulfur atoms at hexagonal close-pack hollow sites at a distance of 1.75 Å from the centre of the hollow site. Transport calculations were performed with Artaios beta 020914 using B3LYP/G DUNNING-DZP and LANL2/LANLDZ ECPs according to published methods^46^.

### Data availability

All of the data used to prepare this manuscript and the Supplementary Information are available upon request.

## Additional information

**How to cite this article:** Carlotti, M. *et al*. Conformation-driven quantum interference effects mediated by through-space conjugation in self-assembled monolayers. *Nat. Commun.* **7,** 13904 doi: 10.1038/ncomms13904 (2016).

**Publisher's note:** Springer Nature remains neutral with regard to jurisdictional claims in published maps and institutional affiliations.

## Supplementary Material

Supplementary Information Supplementary Figures, Supplementary Tables, Supplementary Notes, Supplementary Methods and Supplementary References.

## Figures and Tables

**Table 1:** Summary of SAMs characterization and Au^TS^/SAM//EGaIn junctions properties.

**Compound**	**AH**	**AC**	**PCP**	**OPE3**	**SC18**
XPS thickness (Å)	23.9 (19.0*)	16.2 (25.1*)	15.8	13.7 (17.5†)	20.9
Ellipsometric thickness‡ (Å)	25.4±0.5	17.0±0.5	14.6±0.4	14.3±0.6 (20.6†)	20.2±0.5
Density (10^14^ molecules per cm^2^)	4.7	4.6	3.9	3.5	4.6
log|*J*|@0.5 V (A cm^−2^)	−3.37±0.84	−2.18±0.44	−3.63±0.80	−2.27±0.30	−4.96±0.87
Yield of working junctions (%)	96	98	94	92	79
Number of working junctions	55	56	75	60	28
Number of traces	550	560	750	600	280

AC, 2,6-bis(((4-acetylthio)phenyl)ethynyl)antracene; AH, 2,6-bis(((4-acetylthio)phenyl)ethynyl)-9,10-dihydroantracene; OPE3, 1,4-bis(((4-acetylthio)phenyl)ethynyl)benzene; PCP, pseudo-p-bis((4-(acetylthio)phenyl)ethynyl)-p-[2,2]cyclophane; SC18, CH_3_(CH_2_)_17_S; XPS, X-ray photoelectron spectroscopy.

^*^From ref. 9.

^†^From ref. 19.

^‡^Using *n*=1.5.
