# Supplementary material for: Conformation-driven quantum interference effects mediated by through-space conjugation in self-assembled monolayers
Source: Nat Commun. 2016 Dec 20;7:13904. doi: 10.1038/ncomms13904 (PMC5187444; doi:10.1038/ncomms13904)
Supplement: Supplementary Information — Supplementary Figures, Supplementary Tables, Supplementary Notes, Supplementary Methods and Supplementary References. [file ncomms13904-s1.pdf]

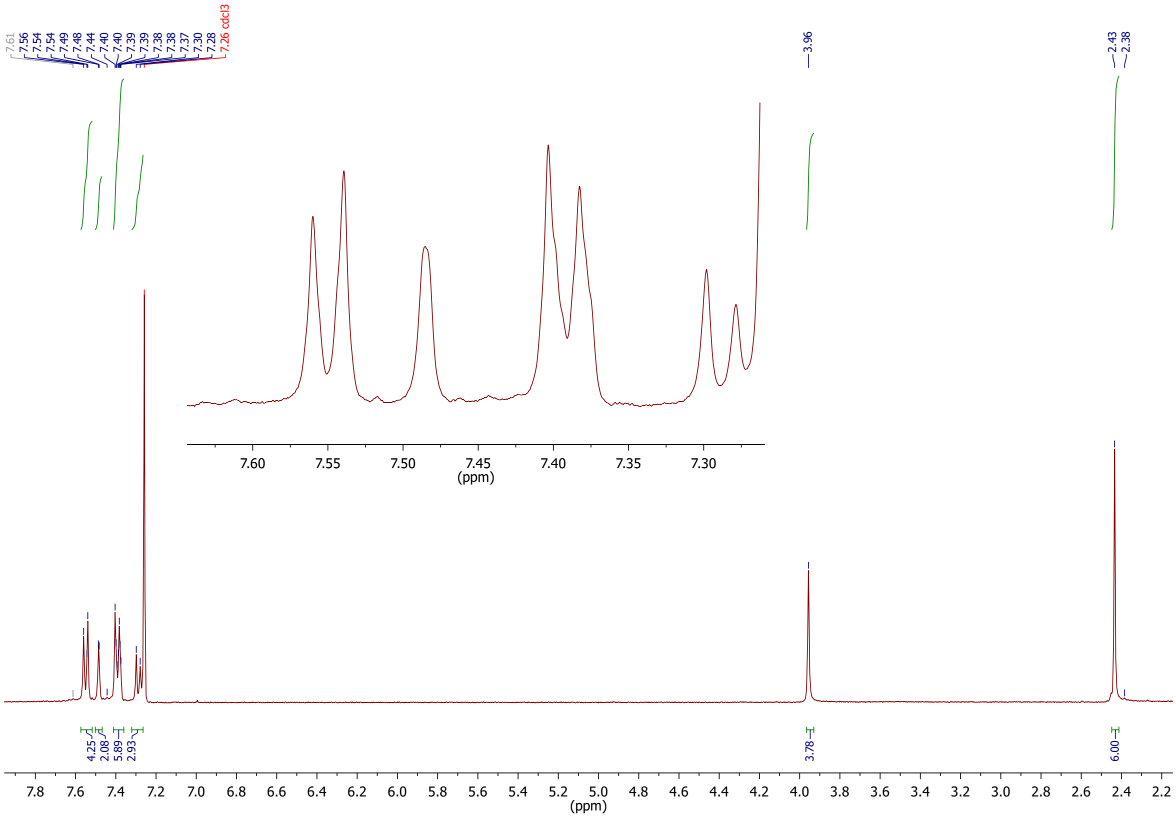

Supplementary Figure 1. ^{1}**H-NMR spectra of AH (400 MHz, $CDCl_3$) recorded at ambient temperature. The single singlet at 3.96 ppm for the hydrogen atoms in the 9 and 10 positions, indicates that at this temperature AH-planar and AH-bent are in equilibrium and interconvert faster than the NMR timescale, that is, in agreement with the calculations in Table 1.**

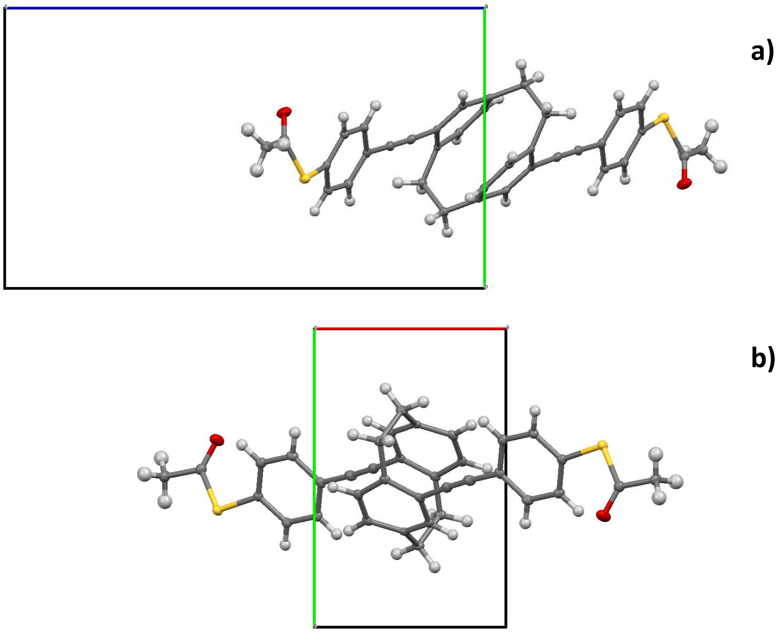

Supplementary Figure 2. Crystal structure for PCP. a) View along a-axis; b) view along c-axis. Color code: C, grey; H, white; S, yellow; O, red.

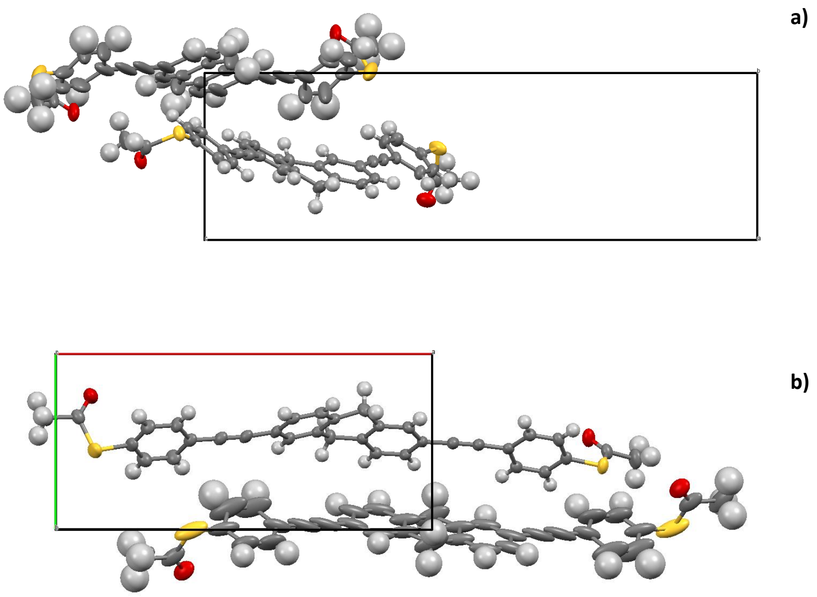

Supplementary Figure 3. Crystal structure for AH. a) View along a-axis; b) view along c-axis. Color code: C, grey; H, white; S, yellow; O, red.

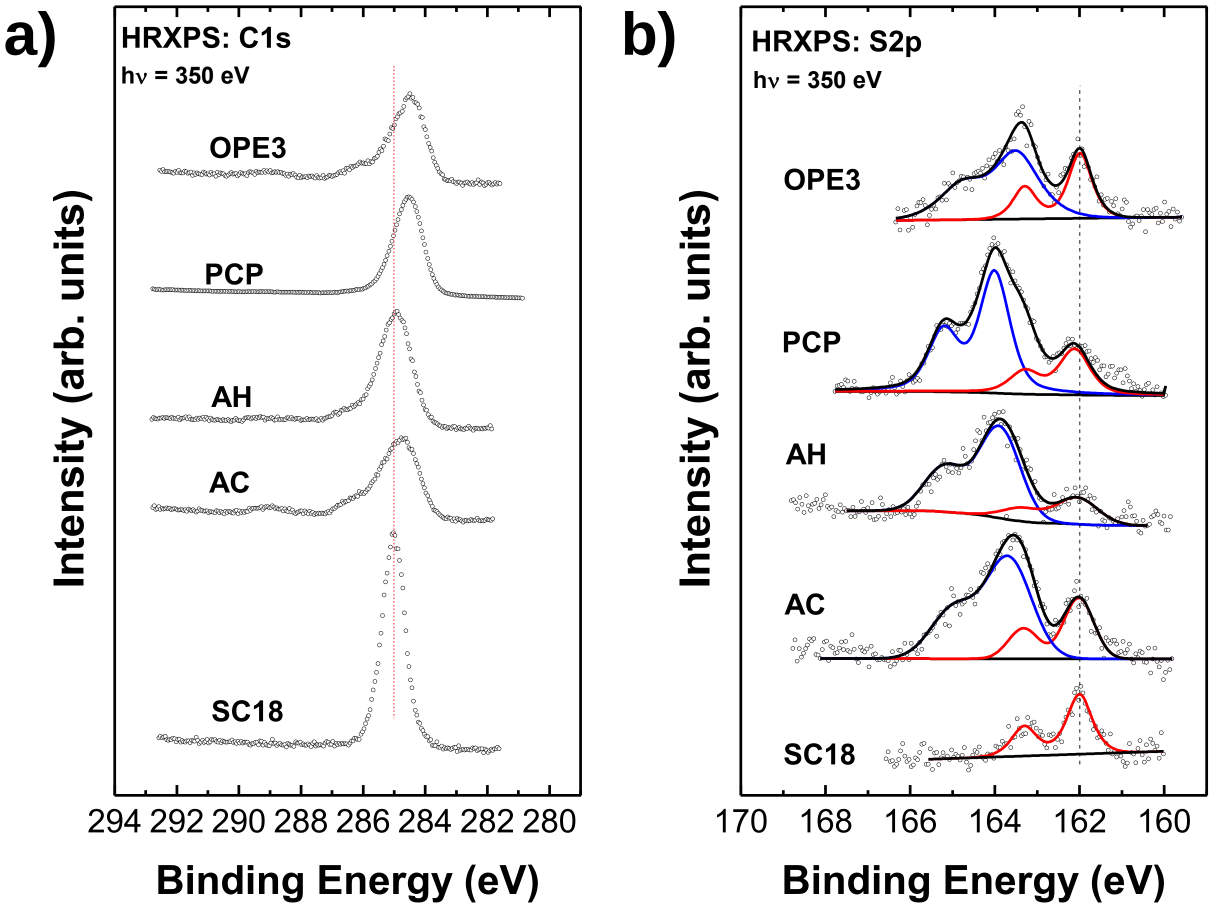

Supplementary Figure 4. C 1s (a) and S 2p (b) HRXP spectra of the SAMs studied as well as reference SC18 monolayer. The spectra were acquired at a PE of 350 eV. The peaks in the S 2p spectra are decomposed into individual doublets associated with the thiolate headgroup at the Au/SAM interface and a -SAc group at the SAM/ambient interface (see text for details).

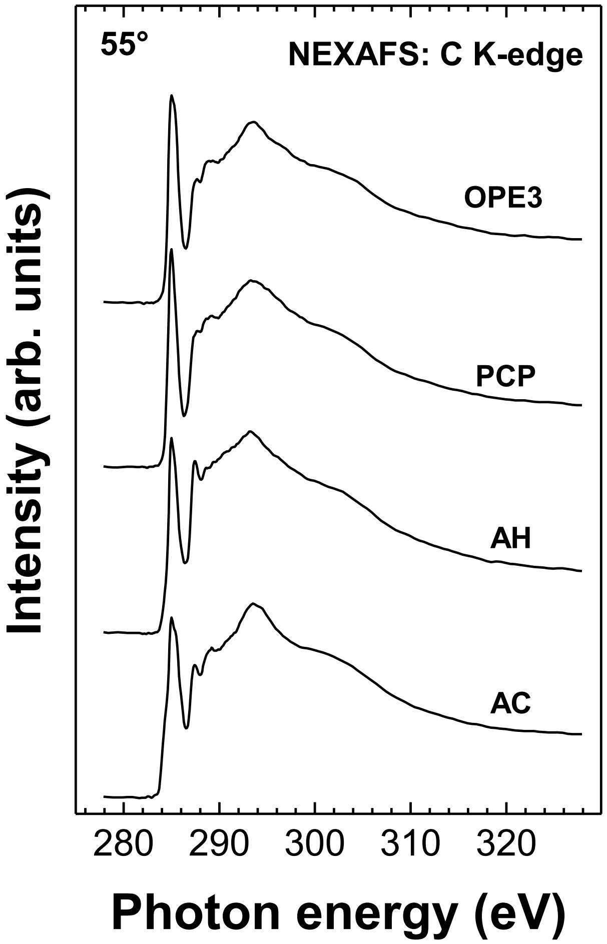

Supplementary Figure 5. C K-edge NEXAFS spectra of the SAM studied. The spectra were measured at an X-ray incidence angle of 55° and are exclusively representative of the electronic structure of the monolayers.[1] The spectra exhibit the characteristic shape and the characteristic absorption resonances of the OPE compounds,[2] above all the slightly asymmetric, joint p* resonance at approximately 285.0 eV. No traces of contamination, above all a very pronounced resonance of carboxyl (most frequent contamination) at 288.8 eV,[3] are observed, revealing a purity of the monolayers.

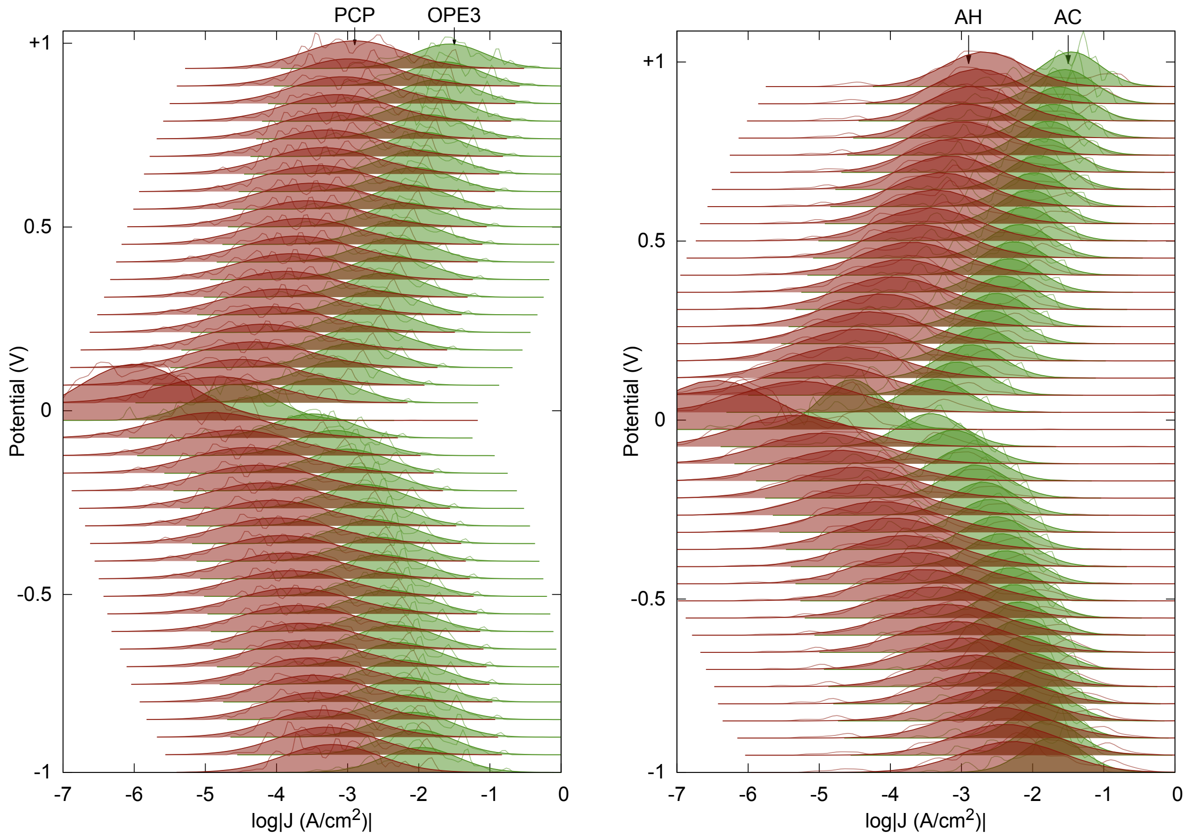

Supplementary Figure 6. Pseudo-3D plots of the raw data showing histograms (solid lines) and Gaussian fits (filled curves) of all of the J/V data for, right: AC (red) and AH (green), left: OPE (red) and PCP (green). The pseudo-Y axis corresponds to the potential for each histogram and the X-axis are the bins in units of $log|J(A/cm^2)|$, that is, the plot resembles a J/V rotated by $90°$.

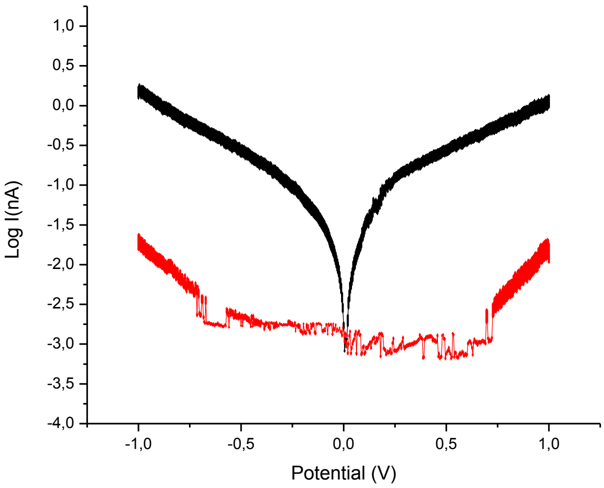

Supplementary Figure 7. Semilog plot of I vs. V for CP-AFM junctions comprising AC (black) and AH (red). The error bars are calculated using α= 0.95 and n= 40.

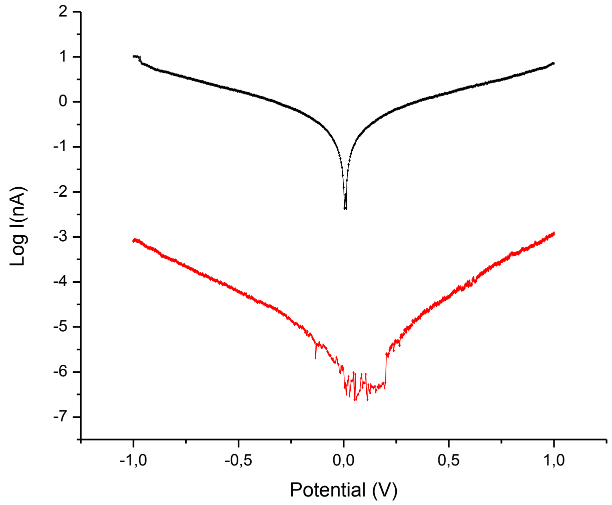

Supplementary Figure 8. Semilog plot of I vs. V for CP-AFM junctions comprising OPE3 (black) and PCP (red). The error bars are calculated using α= 0.95 and n= 40.

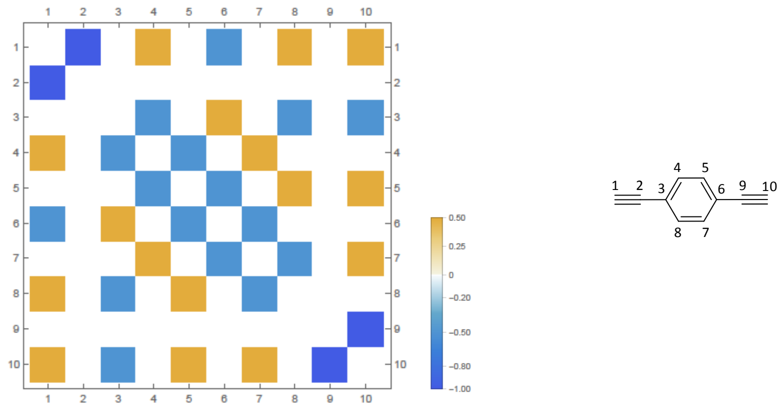

Supplementary Figure 9. Colored representation of the $G(s, r)$ matrix for OPE3. White positions represent zero-elements ascribable to quantum interference in the path trough the atoms labeled r and s. Numbering of the molecule is presented on the right side of the figure.

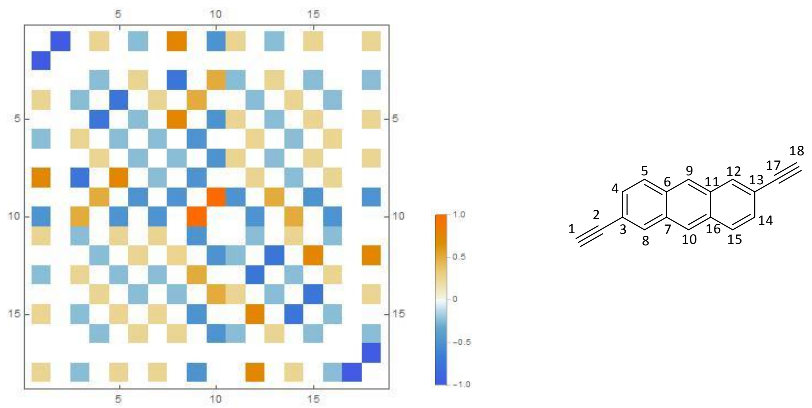

Supplementary Figure 10. Colored representation of the $G(s, r)$ matrix for AC.White positions represent zero-elements ascribable to quantum interference in the path trough the atoms labeled r and s. Numbering of the molecule is presented on the right side of the figure.

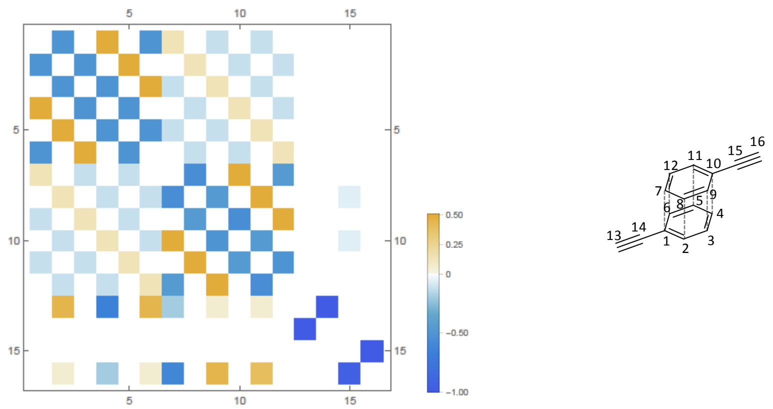

Supplementary Figure 11. Colored representation of the $G(s, r)$ matrix for PCP. White positions represent zero-elements ascribable to quantum interference in the path trough the atoms labeled r and s. Dashed lines deplict through-space interaction (see main text). Numbering of the molecule is presented on the right side of the figure.

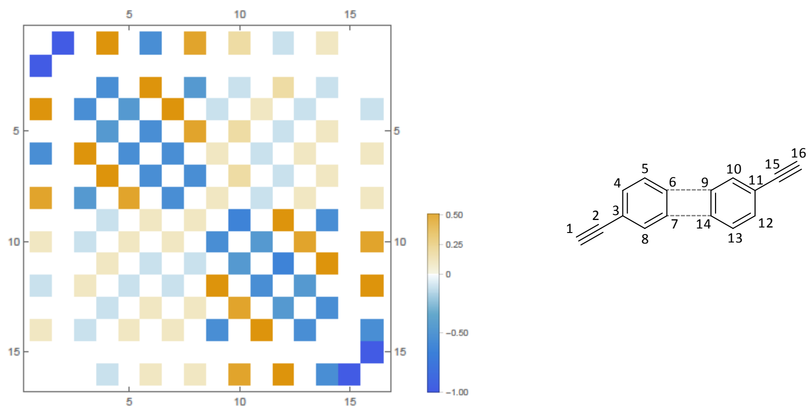

Supplementary Figure 12. Colored representation of the $G(s,r)$ matrix for AH. White positions represent zero-elements ascribable to quantum interference in the path trough the atoms labeled r and s. Dashed lines deplict through-space interaction (see main text). Numbering of the molecule is presented on the right side of the figure.

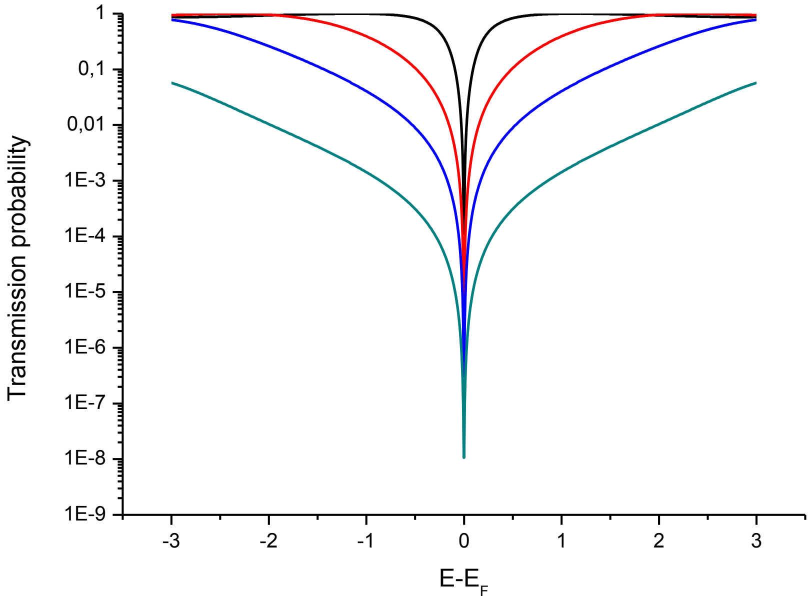

Supplementary Figure 13. Transport probability vs. energy of the PCP-like model presented in figure 11 using four different values of α: -2.0 eV (black line), -1.1 eV (red line), -0.5 eV (blue line), and -0.1 eV (green line).

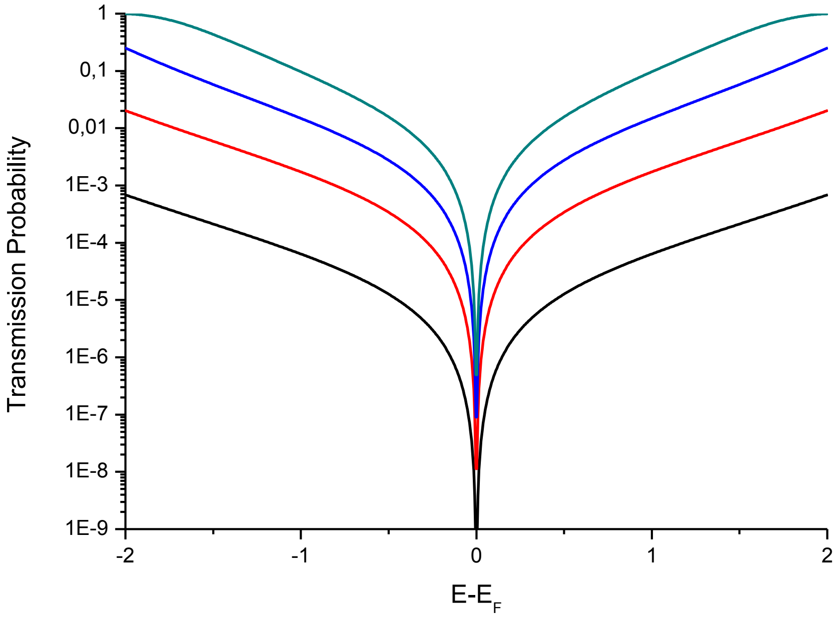

Supplementary Figure 14. Transport probability vs. energy of the AH-like model presented in figure 12 using four different values of α: -2.0 eV (black line), -1.1 eV (red line), -0.5 eV (blue line), and -0.1 eV (green line).

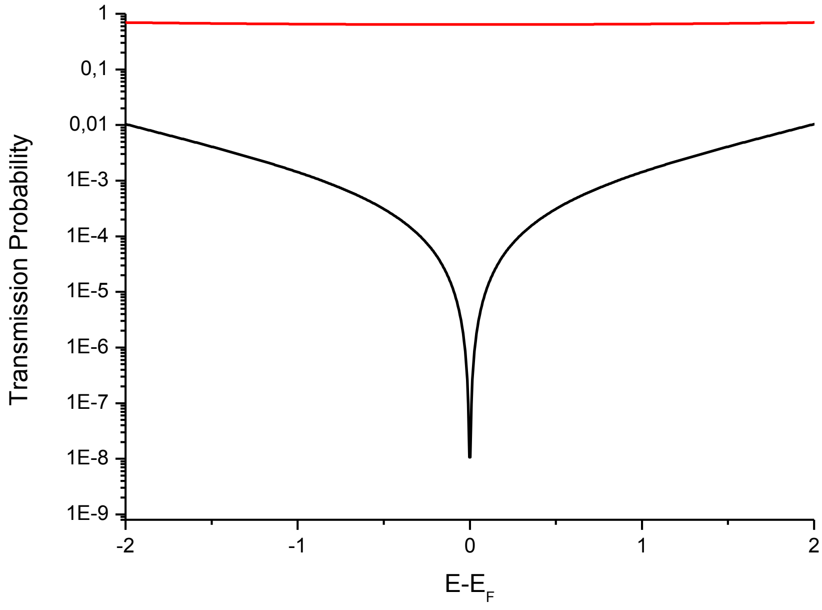

Supplementary Figure 15. Transport probability vs. energy of the PCP -like model presented in figure 11 (red line) and the OPE3 core presented in figure 9 (black line). PCP spectra was calculated assuming α= 0.5 eV between non-nearest neighbors interactions.

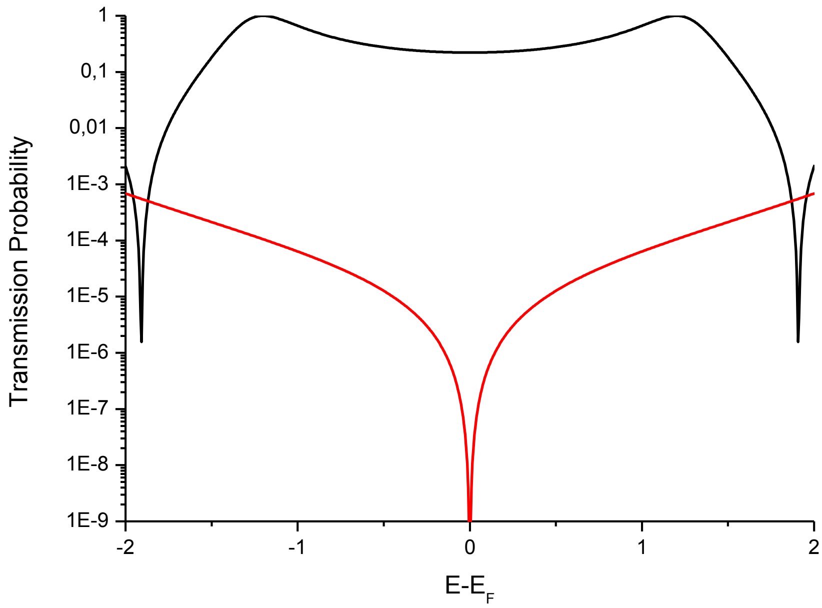

Supplementary Figure 16. Transport probability vs. energy of the AH-like model presented in figure 12 (red line) and the AC core presented in figure 10 (black line). AH spectra was calculated assuming α= 0.5 eV between non-nearest neighbors interactions.

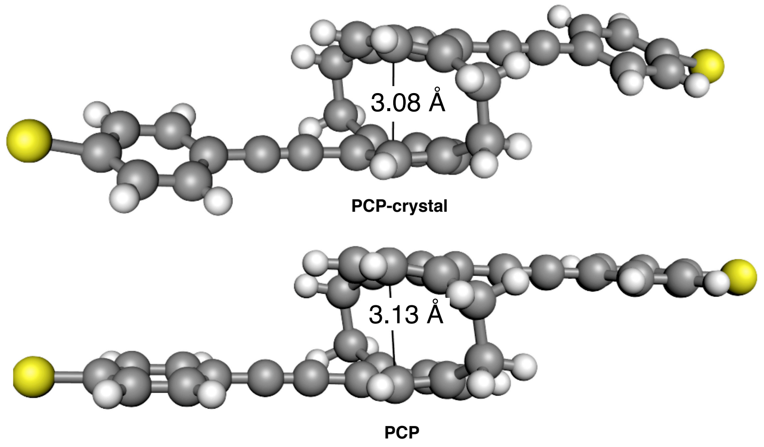

Supplementary Figure 17. Ball-and-stick models of PCP and PCP-crystal showing slight difference in through-space distances and the more pronounced differences in the phenylacetylene groups.

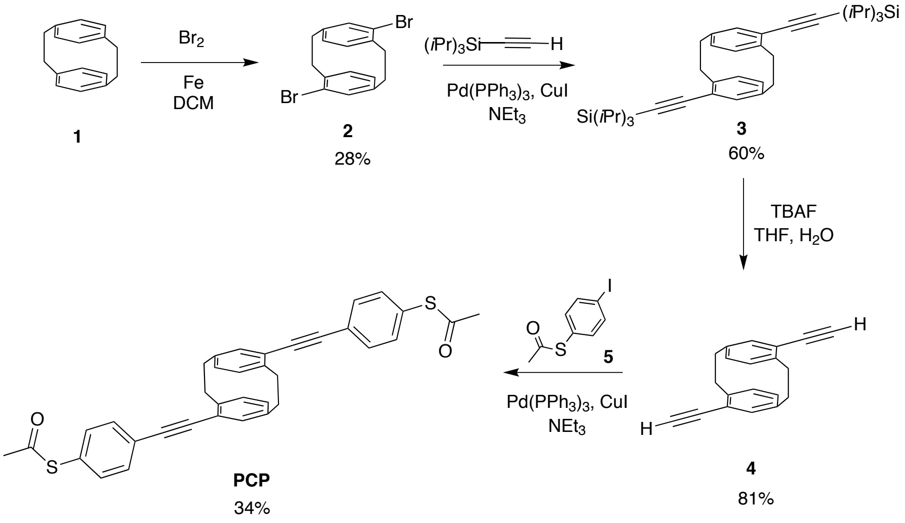

Supplementary Figure 18. Reactions scheme for PCP.

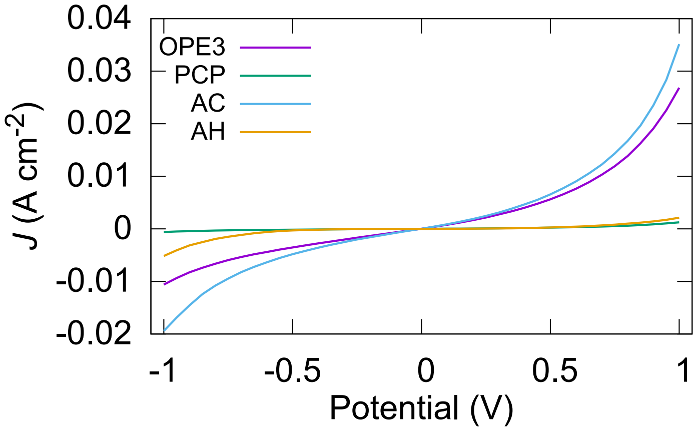

Supplementary Figure 19. Linear plots of J versus V for OPE3, PCP, AC, and AH showing a trend in J: AC > OPE3 > AH > PCP.

Supplementary Table 1. Summary of thermochemistry calculations.

Conformer	**AH Bent**	**AH Planar**
Gibbs Free Enthalpy (eH)	−1154.30718410	−1154.31137303
Electronic Energy (eH)	−1154.65970157	−1154.66209810
Zero Point Energy (eH)	0.39592210	0.39676337
Entropy (eH)	0.06230832	0.06631054
Energy Correction (eH)	0.00283112	0.00283112
Enthalpy Correction (eH)	0.00094373	0.00094373
**Total Gibbs Free Enthalpy Change**		
ΔH (kcal/mol)	2.62856614	
**Total Energy and Entropy Change**		
Δ Electronic Energy (kcal/mol)	1.50382976	
Δ Zero Point Energy (kcal/mol)	-0.52789945	
Δ Energy Correction (kcal/mol)	0	
Δ Enthalpy Correction (kcal/mol)	0	
$-T\Delta S$ @298K (kcal/mol)	2.51140506	
ΔG (kcal/mol)	3.49	

Supplementary Table 2. Crystallographic data for PCP and AH.

Compound	PCP	AH
Chemical formula	$C_{36}H_{28}O_2S_2$	$C_{34}H_{24}O_2S_2$
M_r	556.70	528.65
Crystallographic System	monoclinic	monoclinic
Color, Habit	colorless, block	yellow, block
size (mm)	0.48x0.14x0.14	0.24x0.22x0.07
Space Group	P2(1)/n	P2(1)/n
a (Å)	6.9148(3)	18.345(3)
b (Å)	10.7585(5)	8.1486(11)
c (Å)	18.4018(10)	28.253(4)
β (deg)	90.9049(16)	107.351(4)
V (Å^3)	6.9148(3)	18.345(3)
Z	2	8
a (Å)	6.9148(3)	18.345(3)
ρ_{calc} (g/cm^3)	1.351	1.307
μ (Mo K$^\alpha$, (cm^{-1})	0.228	0.228
F(000)	584	1656
T (K)	100(2)	100(2)
θ range (deg)	3.54 - 27.10	2.85 - 26.37
Data collected (h,k,l)	-8:7, -13:13, -23:23	-22:22, -10:10, -35:35
no. of reflections collected	23998	64871
no. of independent reflections	3017	8227
observed reflections	2753 ($F_o \geq 2\sigma(F_o)$)	5775 ($F_o \geq 2\sigma(F_o)$)
R(F) (%)	3.49	8.99
wR(F^2) (%)	9.66	25.11
GooF	1.049	1.027
Weighting a,b	7 0.0537, 0.8240	0.0986, 11.1329
Parameters refined	182	517
restraints	0	0
Residual density min, Max	-0.241, 0.536	-1.107, 1.046

Supplementary Table 3. V_{trans} values and standard deviations and DFT frontier orbital energies for Au^{TS}/SAM//EGaIn junctions

SAM	V^+_{trans} [V]	V^-_{trans} [V]	HOPS[a] [eV]
AC	0.71 ± 0.08	-0.82 ± 0.10	-5.5713
AH	0.29 ± 0.08	-0.26 ± 0.07	-5.5452
			-5.5599[b]
OPE3	0.78 ± 0.05	-0.89 ± 0.05	-5.5941
PCP	0.63 ± 0.16	-0.86 ± 0.07	-5.6711
			-5.5885[b]

[a]Highest occupied π state.

[b]Crystal structure.

Supplementary Note 1: The Role of the Thickness of Self-Assembled Monolayers in Tunneling Charge-Transport.

Tunneling charge transport can be described using an approximately rectangular barrier where the width represents the tunneling distance and the height the energy barrier. Tunneling charges take advantage of molecular states that exist below that barrier. States that allow transport are open channels and the more open channels there are, the higher the conductance. Open channels exist at zero bias, but more are recruited with increasing bias until, eventually, the Fermi level E_f aligns with the barrier and transmission goes to unity. There are three possible pathways for tunneling transport through a self-assembled monolayer (SAM); i) Via intermolecular states created by the close contacts between molecules. ii) Via molecular states, but traversing adjacent molecules instead of along individual molecules. iii) Via individual molecules, along their backbones. In cases i and ii the thickness of the SAM defines the width of the tunneling barrier. As is discussed in the Main Text, there is little evidence supporting that model and plenty supporting case iii. In case iii, the wavefunction describing a state near E_f simply has non-zero amplitude on both sides of the junction. However, due to the uncertainty and variability in the experimentally determined thickness of SAMs (shown in Table 1 of the Main Text,) it is useful to examine the possibility that that the differences in conductance reported are due to the differences in thickness.

Tunneling through a rectangular barrier, as described above, follows the equation $J = J_0 \exp(-\beta d)$. Thus, assuming the same value of β, the ratio of distances between two junctions d_a, d_b can be written according to Supplementary Eq. 1 . Using a value of $J_0 = 0.001$ Acm^{-2} for conjugated molecules in EGaIn junctions taken from Ref 4 and values of J for AH and AC from Table 1 of the Main Text, $\chi = 0.64$ (where the subscript a is AC and b is AH,) reflecting the lower observed conductivity of AH relative to AC. Using the thickest value for d of AH (from ellipsometry) and the thinnest value of AC that ratio is indeed 0.64. Using the thinnest value of AH and the thickest value of AC (both from XPS) the ratio is 1.32. Thus, if we assume that the barrier width is defined by the thickness of the SAMs, the total range of χ is $0.64 - 1.32$ and does not predict which SAM will be more conductive; the numerical agreement of the lower bounds is coincidence. If, instead, we assume that the width of the barrier is defined by the end-to-end distances of the molecules $d_a = 24.62$ Å for AC and $d_b = 24.46$ Å for planar AH (the longest possible distance) and $\chi = 1.00$, predicting no difference in conductivity. Using the bent form of AH (the shortest possible distance) $d_b = 23.52$ Å, $\chi = 1.05$, predicting that AC is slightly less conductive than AH. The same analysis comparing OPE3 and PCP gives a range of χ of $0.87 - 1.41$ from thickness. The S-S distance of OPE3 is $d_a = 24.46$ Å and PCP is $d_b = 24.62$ Å for which $\chi = 0.99$. Computed from the values of J, $\chi = 0.42$. Thus, the experimental thickness do not predict which SAM is more conductive for either AH and AC or OPE3 and PCP. Given this fact and the general observation that transport is dominated by molecular length, generally, we conclude that, in the absence of Quantum Interference effects, it is reasonable to assume that the conductivities of each pair of SAMs would be nearly identical. Supplementary Figure 19 shows the conductance data plotted on a linear scale where is is readily apparent that the conductivities of OPE3 and AC are considerably higher than PCP and AH.

$$\chi = \frac{d_a}{d_b} = \frac{\ln |J_a/J_{0a}|}{\ln |J_b/J_{0b}|} \tag{1}$$

Supplementary Note 2: Characterization of Self-Assembled Monolayers.

The AH, AC, PCP, and OPE3 SAMs were characterized by ellipsometry, high-resolution X-ray photoelectron spectroscopy (HRXPS), and angle-resolved near-edge X-ray absorption fine structure (NEXAFS) spectroscopy. Ellipsometric measurements were acquired in air on a V-Vase Rotating Analyzer equipped with a HS-190 monochromator acquired from J. A. Woollam Co., Inc.. The samples were measured over a range from 300 nm to 800 nm with an interval of 10 nm at 65°, 70° and 75° angle of incidence. The optical parameters for the 100 nm gold layer were obtained by measuring a freshly prepared template stripped Au surface. The thickness of the SAMs was obtained by fitting the data with a two-layer model consisting in one Au layer (with the determined optical constants) and a Cauchy layer on top described by the parameters A=1.5, B=C=0. For every molecule, at least two samples were analyzed and every sample was measured in at least three different spot. The data presented in the maint text are the calculated average value. The errors were always <5 % of the mean value.

The HRXPS measurements were carried our at the synchrotron storage ring MAX II at MAX-IV facility in Lund, Sweden, using the bending magnet beamline D1011 and an experimental station equipped with a SCIENTA SES200 electron energy analyzer and a partial electron yield (PEY) detector. The NEXAFS spectroscopy measurements were performed at the HE-SGM beamline (bending magnet) of a German synchrotron radiation facility, BESSY II in Berlin. A custom-designed experimental station was used.[5] All experiments were performed at room temperature and under ultra high vacuum conditions at a base pressure of $<1.5 \times 10^{-9}$ mbar. Special care was taken to avoid X-ray damage during the spectra acquisition.[6]

The HRXP spectra were recorded in the Au $4f$, S $2p$, and C $1s$ regions; the O $1s$ region was monitored as well. The spectra acquisition was performed in normal emission geometry and at photon energies of either 350 or 580

eV. The binding energy (BE) scale of every spectrum was individually calibrated using the Au $4f_{7/2}$ emission line of alkanethiolate covered Au substrate at 84.0 eV.[7] The energy resolution was better than 70-100 meV, which is noticeably smaller than the full width at half maximum (fwhm) of the emissions measured. The spectra were fitted by symmetric Voigt functions and either Shirley-type or linear background. To fit the S $2p_{3/2,1/2}$ doublet, we used two peaks with the same fwhm, the standard[7] spin-orbit splitting of ~ 1.18 eV (verified by fit), and a branching ratio of 2 ($S2p_{3/2}/S2p_{1/2}$). The fits were performed self-consistently: the same fit parameters were used for identical spectral regions.

The intensity values derived within the fitting procedure were used to calculate the effective thicknesses of the SAMs studied. They were estimated on the basis of the C $1s$/Au $4f$ intensity ratio,[8] assuming a standard exponential attenuation of the photoelectron signal[9] and using the attenuation lengths typical of densely packed SAMs[10]. The spectrometer specific constants were determined using the octadecanethiol (SC18) monolayer of well-defined thicknesses as a reference. In addition, molecular packing densities in the SAMs studied were calculated, based on the S $2p$/Au $4f$ intensity ratio, using the same assumptions as in the case of the C $1s$/Au $4f$ evaluation. Only the part of the S $2p$ signal related to the thiolate was used. The SC18 monolayer served as a reference; it has a molecular density of $4.63 \times 10^{14}\,\mathrm{cm}^{-2}$, which corresponds to an area per molecule of 21.6 Å^2.[11]

The NEXAFS spectra were acquired at the C K-edge in the PEY acquisition mode with a retarding voltage of 150 V, respectively. Linear-polarized synchrotron light with a polarization factor of 91 % was used. The energy resolution was approximately 0.3 eV. The incidence angle of the primary X-ray beam was varied from 90° (E vector in surface plane) to 20° (E vector nearly parallel to surface normal) in few steps to monitor the orientational order in the SAMs. This approach is based on the dependence of the cross-section of the resonant photoexcitation process on the orientation of the electric field vector of the synchrotron light with respect to the molecular orbital of interest (so-called linear dichroism in X-ray absorption).[1] Raw NEXAFS spectra were normalized to the incident photon flux by dividing a spectrum of a clean, freshly sputtered gold sample and, subsequently, were reduced to the standard form by subtracting linear pre-edge background and normalizing to the unity edge jump (determined by a nearly horizontal plateau 40 eV to 50 eV above the respective absorption edges). The photon energy scale was referenced to the most intense π^* resonance of highly oriented pyrolytic graphite at 285.38 eV.[12]

Supplementary Note 3: Greens Function Analysis.

In addition to the transmission spectra and the graphical analyses shown above, we wanted to verify that any analysis–done properly–will predict destructive QI. These tests are particularly useful for two reasons 1) PCP did not show experimental evidence for QI outside of the suppressed values of J and 2) QI has not previously been predicted for the type of edge-on arrangement in AH. We begin with the so-called Greens function analysis.

The transmission probability spectra of a lead-molecule-lead system can by calculated by Eq. 2

$$T(E) = Trace[\mathbf{\Gamma}_L \mathbf{G} \mathbf{\Gamma}_R \mathbf{G}^T] \tag{2}$$

where $\mathbf{G}$ is the nonequilibrium Green's function matrix and $\mathbf{\Gamma}_{L/R}$ is the broadening function matrix for the respective elctrodes.[13] Tsuji et al. showed that a qualitative discussion about the molecule transport properties can be limited to a simple approximation of the Green's function at the Fermi energy of the system (assuming weak coupling between the molecules and the electrodes) shown in Eq. 3.[14]

$$\mathbf{G}(E_F) \approx \mathbf{G}^{(0)}(E_F) = [(E_F + i\eta)\mathbf{I} - \mathbf{H}]^{-1} \tag{3}$$

where $\mathbf{I}$ is the unit matrix, $\mathbf{H}$ is the Hückel Hamiltonian matrix and η is a infinitesimal positive number. Assuming that E_F doesn't correspond to any eigen-energies of $\mathbf{H}$ and referring it to 0 ($E_F = 0$), the so-called 0th-order Green's function matrix is obtained, with its elements given by Eq. 4.

$$G(r, s) \approx [-\mathbf{H}^{-1}]_{r,s} \tag{4}$$

By "contacting" the molecule in the positions noted by r and s along the molecule's conjugation path, information about the molecular transport can be obtained from $G(r, s)$. In particular a 0-element in the latter represents a channel in which the transport is not permitted by quantum interference, while a non-0 equals to an open channel. As suggested in Eq. 2, the transport properties depend as well on the broadening function matrices, which describe the interaction between the molecule and the electrodes: for reasons of simplicity, in our Green's function analysis we consider that the matrices $\mathbf{\Gamma}_{L/R}$ have only one non-zero element each and therefore the transmission probability will be proportional to the $G(r, s)$ matrix elements.[14] We therefore calculated $G(r, s)$ for the cores of OPE3, AC, PCP and AH as described by 4. To keep our analysis as simple as possible, we constructed the $\mathbf{H}$ matrix using the energy units of the resonance integral which describes the interaction between two adjacent π orbitals, in this case all the near-neighbor interactions carry the value 1 while all the others can be set to 0. This approach ignores features like different bond distances and neighbors interactions,

which, anyway, do not change the output of a qualitative approach.

The $G(r, s)$ matrix obtained for OPE3 is reported in Fig. 9. Clearly, if we chose r=1 and s=10 - which are the positions to which the phenylthioacetate binsing units are connected -, $G(1, 10) = 1/2$ and no quantum interference is predicted. This case is similar to AC for which the matrix element on going from one end to the molecule (r=1) to the other (s=18) is non-zoero (Fig. 10).

The case is different when considering PCP and AH. In fact, the broken conjugation path prevents the building of the **H** matrix with the previous approximations. To face this problem, we decide to treat trough-space conjugated π-systems as if the sp^2 carbons belonging to the separated π-systems were interacting as adjacent entities but with a lower overlap resonance integral due to the longer spacial separation. Extended formulas to calculate the overlap integrals between atomic orbitals of two interacting atoms were proposed by Mulliken et al.[15]. For PCP the carbons in the stacked phenyl rings were assumed to interact trough-space with their eclipsed neighbor on the other ring. This is represented by the dashed lines in the molecular scheme in Fig. 11. The resulting $G(r, s)$ matrix (Fig. 11) predicts destructive quantum interference on going from r=13 and s=16, in opposition to what observed for OPE3 over a similar path. In the case of AH we assumed no contribution from the sp^3 carbons in the anthracene positions 9 and 10. The resulting molecular scheme and $G(r, s)$ matrix are represented in Fig. 12, where again dashed lines are used to indicate through-space neighbors interactions. Like the previous case of PCP and OPE3, also in the case of AH a destructive interference is observed comparing it to the results obtained for AC. Anyway, if a role of the saturated carbons in the transport is considered (for example as a consequence of hyperconjugation) then the interference is expected to vanish and the matrix for AH to resemble that of AH.

Supplementary Note 4: Tight-Binding/Hückle Model.

In the main text it was observed that even slight changes in the relative positions of the conjugated systems can affect the charge transport in a molecular junction. This result was obtained calculating the transport curves from DFT-dervied Hamiltonians (see Section 1.2) which take in account all the interactions between the atomic orbitals in the molecule and with the leads. However, here we show how qualitatively similar results can be obtained even using a much simpler through-bound model to perform transport calculation. We therefore constructed two topological models for the PCP and AH cores using two phenyl rings positioned face to face or edge to edge respectively and extending the conjugated system with two more carbons to simulate the effect of ring substitution in the calculation (see figures 11 and 12). For simplicity, all the carbon atoms were approximated by a single Slater orbital whose energy was set to 0 eV. For the same reason, the coupling energy derived from the orbital overlap was set to $\alpha = -2.7$ eV for the nearest neighbors (that is, two carbon atoms connected by a bond). The leads, consisting in infinite Au chains, were coupled to the previously mentioned two carbon atoms connected to the rings with a coupling magnitude of 0.5 eV. The results of a charge transport calculation on simple conjugated systems like the OPE3 and AC core with these parameters can be observed in figure 15 and 16.

The through-space interaction in the PCP-like and AH-like systems, which is weaker than the trough-bond one, was expressed by a lower α between a carbon and its nearest neighbor in the other systems. Such interactions are pictured as dashed lines in figures 11 and 12.

To investigate the effect on the transport probability of the entity of the coupling between the two separated systems different values of α ranging from -0.1 eV to -2 eV were used in the calculations. The results for PCP and AH are showed in figures 13 and 14 respectively. Noticeably in both cases, increasing the coupling between the two rings, affects the depth of the interference feature but does not shift it to different energies.

Supplementary Methods

Synthesis of new compounds
***pseudo-p*-bis((4-(acetylthio)phenyl)ethynyl)-*p*-2,2-cyclophane** (PCP) was prepared starting from *p*-2,2-cyclophane (**1**) according to Supplementary Figure 18. *p*-2,2-cyclophane (99%), (Triisopropylsilyl)acetylene (97%), tetrabutylammonium fluoride (TBAF, 1M in THF, 5% H_2O), pipsyl chloride (95%), *N,N*-dimethylacetamide (99.8%) and dichlorodimethylsilane ($\geq$ 98.5%) were purchased from Sigma-Aldrich and used as received. $Pd(PPh_3)_3$ and CuI were purchased from ACROS Organics and stored under nitrogen in the dark at 4 °C. NEt_3 was distilled over CaH. Tetrohydrofuran (THF) was stirred for 1 h in basic alumina (Merck Millipore, 90 active) to remove the stabilizer. The rest of the solvents were used as received. ^{1}H-NMR and ^{13}C-NMR spectra were recorded on a Varian AMX400 (400 MHz) and a Varian VXR-300 (300 MHz) at room temperature. All the spectra were referenced to the solvent line of $CDCl_3$ relative to tetramethylsilane (H, 7.26 ppm; C, 77.0 ppm). FT-IR spectra were recorded on a Nicolet Nexus spectrometer using the SMART iTR for ATR measurement (diamond).

***pseudo-p*-dibromo-*p*-2,2-cyclophane (2)**. The synthesis of **2** was based on a literature method.[16] Under inert atmosphere (N_2), 11,552 g of **1** (55.2 mmol) were dissolved in 80 mL of CH_2Cl_2. 160 mg of Fe (2.87 mmol) were added and the reaction mixture was refluxed for 30 minutes. A solution of Br_2 in CH_2Cl_2 was prepared by mixing 7 mL of Br_2 and 40 mL of CH_2Cl_2. The first half was added over a period of 30 minutes, then the reaction mixture was brought to reflux again and the remaining half was added over a period of 12 hours. The reaction was left at reflux for 18 hours in total. The reaction mixture was cooled down and the precipitate filtered off. The solid residue was recrystallized from $CHCl_3$ to yield 5.86 g of desired product as a white solid (28% yield). ^{1}H-NMR (400 MHz, $CDCl_3$) δ 7.14(dd, J=7.9, 1.7 Hz, 2H), 6.51(d, J=1.4 Hz, 2H), 6.44(d, J=7.8 Hz, 2H), 3.49(ddd, J=13.1, 10.4, 2.3 Hz, 2H), 3.16(ddd, J=12.6, 10.3, 4.9 Hz, 2H), 2.94(m, 2H), 2.85(ddd, J=13.2, 10.8, 4.9 Hz, 2H). IR (ATR, cm^{-1}): 2935, 2850, 1584, 1536, 1473, 1390, 1186, 1031.

***pseudo-p*-bis((tri-tert-butylsilyl)ethynyl)-*p*-2,2-cyclophane (3)**. In a flask under inert atmosphere (N_2), 1,00 g of **2** (2.7 mmol), 1,40 mL of (triisopropylsilyl)acetilene (6.4 mmol), 0.172 g of $Pd(PPh_3)_3$ (0.15 mmol) and 0.030 g of CuI (0.15 mmol) were added to 20 mL of NEt_3. The reaction mixture was left at reflux for 22h. The reactor was then cooled and the solvent removed under vacuum. The solid residue was stirred in 100 mL of hot n-hexane and filtered. The solvent was evaporated *in vacuo* and the product purified by recrystallization from n-hexane to obtain 715 mg of white crystals (60% yield). ^{1}H-NMR (400 MHz, $CDCl_3$) δ 7.03(dd, J=7.9, 1.9 Hz, 2H), 6.52(d, J=1.9 Hz, 2H), 6.42(d, J=7.8 Hz, 2H), 3.63(ddd, J=13.0, 10.5, 2.8 Hz, 2H), 3.17(ddd, J=12.8, 10.5, 4.6 Hz, 2H), 2.98(ddd, J=12.8, 11.7, 2.8 Hz, 2H), 2.85(ddd, J=12.8, 10.7, 4.6 Hz, 2H), 1.19 (s, 42H).

***pseudo-p*-diethynyl-*p*-2,2-cyclophane (4)**. 341 mg of **3** (0.6 mmol) were dissolved in 5 mL of THF. 1.8 mL of a TBAF solution 1M in THF (1.8 mmol) were added and the solution left stirring for 20 h. The solvent was then evaporated *in vacuo* and the residue dissolved in 20 mL of CH_2Cl_2 and filtered over silica gel (SiliaFlash P60 Silicycle). The organic phase was then washed with water (3x100 mL) and brine (1x150 mL). The solvent was removed under vacuum and the product collected as a white powder, 124 mg (81% yield). ^{1}H-NMR (400 MHz, $CDCl_3$) δ 7.01(dd, J=8.0, 2.0 Hz, 2H), 6.56(d, J=1.9 Hz, 2H), 6.45(d, J=7.9 Hz, 2H), 3.58(ddd, J=13.2, 10.6, 3.0 Hz, 2H), 3.27(s, 2H), 3.19(ddd, J=12.7, 10.5, 4.4 Hz, 2H), 2.97(m, 2H), 2.87(ddd, J=13.0, 10.7, 4.5 Hz, 2H). IR (ATR, cm^{-1}): 3274, 2928, 2866, 1480, 1432, 1404, 1243, 1047, 897.

(4-iodophenyl)ethanethioate (5). The procedure for the synthesis of this compound was taken from the literature.[17] In a flask under inert atmosphere (N_2), 3.073 g of pipsyl chloride (10.2 mmol) and 2.8 mL of *N,N*-dimethylacetamide (2.63 g, 30,2 mmol) were dissolved in 80 mL of degassed 1,2-dichloroethane. In a different flask under inert atmosphere (N_2), 2.36 g of Zn (36.3 mmol) and 4.3 mL of Cl_2SiMe_2 (4.58 g, 53.3 mmol) were stirred in 80 mL of degassed 1,2-dichloroethane at 60 °C for 30 minutes, then the first solution was added. The reaction mixture was heated to 75 °C and left for 2 h. Absence of pipsyl chloride was checked via TLC (Rf $\sim$ 0.4 in hexane). The solution was then cooled to 45 °C and 0.94 mL of acetyl chloride (1.038 g, 13.2 mmol) were added. The reactor was left at 50 °C for 30 minutes. The reaction mixture was filtered still hot and the filtrate poured in 300 mL of water. The aqueous phase was extracted with dichloromethane (3x150 mL). The organic solvent was evaporated *in vacuo* and the product purified by sublimation (70 °C, $5 \cdot 10^{-4}$ mbar) to obtain 2.420 g of white crystals (83% yield). ^{1}H-NMR (400 MHz, $CDCl_3$) δ 7.74(m, 2H), 7.13(m, 2H), 2.42(s, 3H). IR (ATR, cm^{-1}): 1691, 1464, 1381, 1353, 1119, 1087, 1004.

***pseudo-p*-bis((4-(acetylthio)phenyl)ethynyl)-*p*-2,2-cyclophane (PCP)**. In a flask under inert atmosphere (N_2), 82 mg of **4** (0.32 mmol), 342 mg of **5** (1.32 mmol), 23 mg of $Pd(PPh_3)_3$ (0.02 mmol) and 3.8 mg of CuI (0.02 mmol) were stirred in 8 mL of NEt_3. The system was heated to 70 °C and left for 18 h. The reaction mixture was poured in 100 mL of water which was carefully acidified with HCl 6 M. The aqueous layer was extracted with dichloromethane (3x20 mL). The product was recrystallized twice from dichloromethane to obtain 61 mg of

a slightly-yellow powder (34% yield). ^{1}H-NMR (400 MHz, $CDCl_3$) δ 7.61(m, 4H), 7.44(m, 4H), 7.01(dd, J=7.8, 1.9 Hz, 2H), 6.60(d, J=1.9 Hz, 2H), 6.51(d, J=7.9 Hz, 2H), 3.66(ddd, J=13.2, 10.4, 2.9 Hz, 2H), 3.22(ddd, J=13.1, 10.3, 4.7 Hz, 2H), 3.06(ddd, J=12.9, 11.7, 2.9 Hz, 2H), 2.94(ddd, J=12.4, 10.7, 4.7 Hz, 2H), 2.46(s, 6H). IR (ATR, cm^{-1}): 2935, 1698, 1487, 1409, 1396, 1118, 1089, 948.

X-Ray Crystal Structures

Single crystals of compounds PCP and AH were mounted on top of a cryoloop and transferred into the cold nitrogen stream (100 K) of a Bruker-AXS D8 Venture diffractometer. Data collection and reduction was done using the Bruker software suite APEX2. The final unit cell was obtained from the xyz centroids of 9991 (PCP) or 9896 (AH) reflections after integration. A multiscan absorption correction was applied, based on the intensities of symmetry-related reflections measured at different angular settings (*SADABS*). (Bruker, [2012]; APEX2 [v2012.4-3], *SAIN*T [Version 8.18C] and *SADABS* [Version 2012/1]; Bruker AXS Inc., Madison, Wisconsin, USA.) The structures were solved by direct methods using *SHELXS*, and refinement of the structure was performed using *SHLELXL*.[18] The hydrogen atoms were generated by geometrical considerations, constrained to idealized geometries and allowed to ride on their carrier atoms with an isotropic displacement parameter related to the equivalent displacement parameter of their carrier atoms. For AH, refinement was complicated by disorder: one of the two independent molecules in the unit cell appears to be disordered over an inversion center. Attempts to describe the disordered part by a two-site occupancy model was not satisfactory. As a result of the disorder, the metrical parameters of this molecule are not determined with high accuracy. In addition, the central anthracene core of this molecule is planar which could be an artifact enforced by the inversion symmetry. On the other hand, inspection of the atomic displacement parameters shows that the main disorder is in the plane of the molecule which seems to suggest that the molecule indeed prefers a planar conformation. The second independent molecule is well-defined and clearly shows a bent anthracene core (dihedral angle between the two flanking C6 rings of 19.05). Crystal data and details on data collection and refinement are presented in Supplementary Table 2, unit cells for PCP and AH are presented in Supplementary Figures 2 and 3 respectively.

Preparation of Self-Assembled monolayers

SAMs were formed by incubating the thioacetate precursors with 1x1 cm template-stripped Au surfaces (100 nm-thick) overnight in 3 mL of 50 μ M solution of the respective compound in freshly distilled toluene followed by addition of 0.05 mL of 17 mM diazabicycloundec-7-ene (DBU) solution in toluene 1h prior the measurement. The substrates were then rinsed with ethanol and let to dry for 30 minutes before performing the measurements. The same procedure was followed for the preparation of the Au-on-mica samples (1x1 cm, 200 nm thick Au, obtained from Phasis) used for CP-AFM data. During SAM formation, leaving the metal substrate in contact with the compound solution and the deprotecting agent for longer time increase the risk of multi-layers formation in case of di-SAc derivatives.

J/V Data Processing

Data were acquired as described and then parsed in a "hands-off" manner using Scientific Python to produce histograms of J for each value of V, the associated Gaussian fits (using a least-squares fitting routine) and the conductance heatmap plots. For the heatmap plots, $G = log|\frac{dJ}{dV}|$ was computed from un-smoothed numerical derivatives from which histograms of G for each value of V were constructed. The data in the heatmap plots were interpolated from Gaussian fits to the histograms of G (using a least-squares fitting routine) to provide data for values between experimental values of V. Plots were generated using Gnuplot 5.1.

The semi-log plot of the J/V data in the Main Text are scaled such that the magnitude of J appears to be equal for PCP and AH and for OPE3 and AC. Supplementary Figure 19 shows these data in a linear plot where a trend in the Gaussian average values $\mu_{\log}$ show the trend: AC > OPE3 > AH > PCP. The pairs in this trend oppose both the theoretical molecular lengths and the measured thicknesses of the SAMs; that is, PCP is shorter than AH. This violation of simple length dependence is a manifestation of the complex transmission channels and QI effects in conjugated molecules.

Data Analysis

Conductance data from tunneling junctions must be treated carefully because of the exponential dependence of J on variations in the tunneling distance. A common and effective approach to mitigating the influence of measurement artifacts and separating them from the influence of defects in the tunneling junctions is to fit Gaussian distributions to histograms of log $|J|$ for each value of V to determine $\mu_{\log}$ and then computing the decay coefficient, β, by plotting ln $\mu_{\log}$ against molecular length (in Å or number of carbons).[19] When combined with statistical analysis, the confidence intervals (and confidence bands) can be used to determine if an effect is due to under sampling or if the dependence of $\mu_{\log}$ on length is statistically significant and not due to random chance. The value of $\beta \pm error$ derived from this analysis then becomes meaningful for comparisons against other experimental platforms because it strips away experimental differences and reveals a fundamental property of the series of molecules under investigation. We used this methodology of analyzing histograms of log $|J|$ to construct the J/V curves shown in the main text. The histograms of the raw J/V data and Gaussian fits are shown in Figure 6. Values of V_{trans} were computed from Gaussian fits to histograms of the minimum of each plot of $\ln J/V^2$ versus V^{-1}.

The confidence intervals for $\mu_{\log}$ depicted as error bars in the J/V plots were calculated using Equation 5 where $\sigma_{\log}$ is taken from Gaussian fits, for example Figure 6, $n + 1$ is the number of junctions measured and A is taken from a standard table of t-distributions (2.2626 for $n = 9$ at the 95 % confidence level).

$$\mu_{log} \pm A\frac{\sigma_{log}}{\sqrt{n}} \tag{5}$$

CP-AFM measurements

CP-AFM I-V measurements were performed on a Bruker AFM Multimode MMAFM-2 equipped with a Peak Force TUNA Application Module (Bruker). The SAMs grown on Au-on-mica grown as previously reported, were contacted with an Au-coated silicon nitride tip with a nominal radius of 30 nm (NPG-10, Bruker, resonance frequency: 65 kHz, k: 0.35 N/M) in non-scanning mode with a force of 1.8 nN. The sample was grounded using silver paste to connect it to the grounded sample holder. The AFM tip was biased from -1 V to 1 V and from 1 V to -1 V to record the I-V curve (512 points per trace were taken): a max of 50 trace/re-trace cycles per junction were performed (with a min of 20). After every junction, the tip was retracted, moved to a different spot, and engaged again for a total of 40 junction per sample analyzed. Between different samples the tip was cleaned with oxygen plasma. The data were analyzed with the same software used for EGaIn using the current I instead of the current density. The obtained Log$I(V)$ plots are shown in Figure 7 and 8. For AH and PCP the detection limit of the instrument were reached for the lower biases.

Calculations

Frequencies and thermochemistry were calculated using B3LYP/6-311+G* at 298K. According to frequency calculations (see Table 1), ΔG going from the planar to the bent geometry of AH is accessible at room temperature (~ -3.5 kcal/mol). This result suggests that AH rapidly interconverts between the bent and planar forms under the experimental conditions. In solution, this observation is confirmed by the ^{1}H-NMR spectrum of AH in $CDCl_3$ at 400 MHz. This spectrum is presented in Fig. 1; the multiplicity of the signals in aromatic region, together with the singlet at 3.96 ppm for the protons in the 9 and 10 positions (the methylene bridges) are indicative of a symmetric conformation of AH with an inversion center. As the completely symmetric conformer of AH (planar) is higher in energy than the less symmetric conformer (bent), this spectrum indicates that the two conformers interconvert faster than the NMR timescale. If AH were trapped in the lowest-energy conformer predicted by RI-MP2/DFT, AH-planar, the lack of an inversion center would cause the protons on the different faces to split, leading to a doublet (or multiplet) at ~ 3.96 ppm and would likely lead to more complex splitting in the aromatics from the proximity of the periphery phenyl protons.

References

[1] Stohr, J. *NEXAFS Spectroscopy* (Springer-Verlag Berlin Heidelberg, 1992).

[2] Hamoudi, H., Kao, P., Nefedov, A., Allara, D. L. & Zharnikov, M. X-ray spectroscopy characterization of self-assembled monolayers of nitrile-substituted oligo(phenylene ethynylene)s with variable chain length. *Beilstein journal of nanotechnology* **3**, 12–24 (2012).

[3] Shaporenko, A., Adlkofer, K., Johansson, L. & Tanaka, M. Functionalization of gaas surfaces with aromatic self-assembled monolayers: A synchrotron-based spectroscopic study. *Langmuir* (2003).

[4] Carlotti, M., Degen, M., Zhang, Y. & Chiechi, R. C. Pronounced environmental effects on injection currents in egain tunneling junctions comprising self-assembled monolayers. *The Journal of Physical Chemistry C* **120**, 20437–20445 (2016).

[5] Nefedov, A. & Wll, C. Advanced applications of nexafs spectroscopy for functionalized surfaces. *Surface Science Techniques* (2013).

[6] Zharnikov, M. High-resolution x-ray photoelectron spectroscopy in studies of self-assembled organic monolayers. *J. Electron Spectrosc. Relat. Phenom.* **178–179**, 380–393 (2010).

[7] Briggs, D. *Handbook of X-Ray Photoelectron Spectroscopy C. D. Wanger, W. M. Riggs, L. E. Davis, J. F. Moulder and G. E.Muilenberg Perkin-Elmer Corp., Physical Electronics Division, Eden Prairie, Minnesota, USA, 1979. 190 Pp. 195*, vol. 3 (Heyden & Son Ltd., 1981).

[8] Thome, J., Himmelhaus, M., Zharnikov, M. & Grunze, M. Increased lateral density in alkanethiolate films on gold by mercury adsorption. *Langmuir* **14**, 7435–7449 (1998).

[9] Ratner, B. D. & Castner, D. G. Electron spectroscopy for chemical analysis. In *Surface Analysis - The Principal Techniques*, 47–112 (John Wiley & Sons, Ltd, 1997).

[10] Lamont, C. L. A. & Wilkes, J. Attenuation length of electrons in self-assembled monolayers of n-alkanethiols on gold. *Langmuir* **15**, 2037–2042 (1999).

[11] Schreiber, F. Structure and growth of self-assembling monolayers. *Prog. Surf. Sci.* **65**, 151–257 (2000).

[12] Batson, P. E. Carbon 1 *s* near-edge-absorption fine structure in graphite. *Phys. Rev. B* **48**, 2608–2610 (1993).

[13] Datta, S. *Quantum Transport: Atom to Transistor* (Cambridge University Press, 2005).

[14] Lykkebo, J., Gagliardi, A., Pecchia, A. & Solomon, G. C. Iets and quantum interference: Propensity rules in the presence of an interference feature. *J. Phys. Chem.* **141**, – (2014).

[15] Mulliken, R. S., Rieke, C. A., Orloff, D. & Orloff, H. Formulas and numerical tables for overlap integrals. *The Journal of Chemical Physics* **17**, 1248–1267 (1949).

[16] Reich, H. J. & Cram, D. J. Macro rings. xxxvii. multiple electrophilic substitution reactions of [2.2]paracyclophanes and interconversions of polysubstituted derivatives. *J. Am. Chem. Soc.* **91**, 3527–3533 (1969).

[17] Daniel T. Gryko, . *et al.* Synthesis of porphyrin-linker-thiol molecules with diverse linkers for studies of molecular-based information storage. *The Journal of Organic Chemistry* **65**, 7345–7355 (2000). PMID: 11076590.

[18] Sheldrick, G. M. A short history of *SHELX*. *Acta Crystallographica Section A* **64**, 112–122 (2008).

[19] Reus, W. F. *et al.* Statistical tools for analyzing measurements of charge transport. *J. Phys. Chem. C* **116**, 6714–6733 (2012).
